# *DCAF4*, a novel gene associated with leucocyte telomere length

**DOI:** 10.1136/jmedgenet-2014-102681

**Published:** 2015-01-26

**Authors:** Massimo Mangino, Lene Christiansen, Rivka Stone, Steven C Hunt, Kent Horvath, Dan T A Eisenberg, Masayuki Kimura, Inge Petersen, Jeremy D Kark, Utz Herbig, Alex P Reiner, Athanase Benetos, Veryan Codd, Dale R Nyholt, Ronit Sinnreich, Kaare Christensen, Hisham Nassar, Shih-Jen Hwang, Daniel Levy, Veronique Bataille, Annette L Fitzpatrick, Wei Chen, Gerald S Berenson, Nilesh J Samani, Nicholas G Martin, Sarah Tishkoff, Nicholas J Schork, Kirsten Ohm Kyvik, Christine Dalgård, Timothy D Spector, Abraham Aviv

**Affiliations:** 1 Department of Twin Research and Genetic Epidemiology, King's College London, London, UK; 2 National Institute for Health Research (NIHR) Biomedical Research Centre at Guy's and St. Thomas’ Foundation Trust, London, UK; 3 Epidemiology Unit, The Danish Aging Research Center and The Danish Twin Registry, Institute of Public Health, University of Southern Denmark, Odense, Denmark; 4 Department of Clinical Genetics, and Department of Clinical Biochemistry and Pharmacology, Odense University Hospital, Odense, Denmark; 5 Center of Human Development and Aging, Rutgers, The State University of New Jersey, New Jersey Medical School, Newark, New Jersey, USA; 6 Cardiovascular Genetics Division, Department of Medicine, University of Utah, Salt Lake City, Utah, USA; 7 Department of Anthropology, University of Washington, Seattle, Washington, USA; 8 Center for Studies in Demography and Ecology, University of Washington, Seattle, Washington, USA; 9 Epidemiology Unit, Hebrew University-Hadassah School of Public Health and Community Medicine, Jerusalem, Israel; 10 Department of Epidemiology, University of Washington, Seattle, Washington, USA; 11 Public Health Sciences Division, Fred Hutchinson Cancer Research Center, Seattle, Washington, USA; 12 Department of Geriatrics, Universite de Lorraine INSERM U961, Nancy, France; 13 Department of Cardiovascular Sciences, University of Leicester, Leicester, UK; 14 National Institute for Health Research (NIHR) Leicester Cardiovascular Biomedical Research Unit, Glenfield Hospital, Leicester, UK; 15 QIMR Berghofer Medical Research Institute, Brisbane, Australia; 16 Department of Cardiology, Hadassah University Medical Center, Jerusalem, Israel; 17 Population Sciences Branch of the National Heart, Lung and Blood Institute, Bethesda, Maryland, USA; 18 The Framingham Heart Study, Framingham, Massachusetts, USA; 19 Department of Dermatology, West Herts NHS Trust, Herts, UK; 20 Center for Cardiovascular Health, Tulane University, New Orleans, Louisiana, USA; 21 Department of Genetics, University of Pennsylvania, Philadelphia, Pennsylvania, USA; 22 Department of Molecular and Experimental Medicine, The Scripps Research Institute, San Diego, California, USA; 23 Institute of Regional Health Services Research, University of Southern Denmark, Odense, Denmark; 24 Odense Patient data Explorative Network (OPEN), Odense University Hospital, Odense, Denmark; 25 Institute of Public Health, Environmental Medicine, University of Southern Denmark, Odense, Denmark

**Keywords:** Complex traits, Telomere, cancer: skin, melanoma

## Abstract

**Background:**

Leucocyte telomere length (LTL), which is fashioned by multiple genes, has been linked to a host of human diseases, including sporadic melanoma. A number of genes associated with LTL have already been identified through genome-wide association studies. The main aim of this study was to establish whether *DCAF4* (DDB1 and CUL4-associated factor 4) is associated with LTL. In addition, using ingenuity pathway analysis (IPA), we examined whether LTL-associated genes in the general population might partially explain the inherently longer LTL in patients with sporadic melanoma, the risk for which is increased with ultraviolet radiation (UVR).

**Results:**

Genome-wide association (GWA) meta-analysis and de novo genotyping of 20 022 individuals revealed a novel association (p=6.4×10^−10^) between LTL and rs2535913, which lies within *DCAF4*. Notably, eQTL analysis showed that rs2535913 is associated with decline in *DCAF4* expressions in both lymphoblastoid cells and sun-exposed skin (p=4.1×10^−3^ and 2×10^−3^, respectively). Moreover, IPA revealed that LTL-associated genes, derived from GWA meta-analysis (N=9190), are over-represented among genes engaged in melanoma pathways. Meeting increasingly stringent p value thresholds (p<0.05, <0.01, <0.005, <0.001) in the LTL-GWA meta-analysis, these genes were jointly over-represented for melanoma at p values ranging from 1.97×10^−169^ to 3.42×10^−24^.

**Conclusions:**

We uncovered a new locus associated with LTL in the general population. We also provided preliminary findings that suggest a link of LTL through genetic mechanisms with UVR and melanoma in the general population.

## Introduction

As expressed in leucocytes, telomere length (TL) is a polygenic trait with heritability estimated at 65%.^1^ Genome-wide association (GWA) meta-analyses identified nine loci associated with leucocyte TL (LTL).^2–4^ Of these, at least six harbour genes (*TERC, TERT, NAF1, OBFC1, CTC1* and *RTEL1*) directly related to telomere homeostasis. LTL, which reflects TL in other somatic cells,^5^ is associated with a host of disease. Typically, LTL is short in patients with cardiovascular disease, principally atherosclerosis,^6^ and long in patients with lung adenoma,^7^ ^8^ breast cancer,^9^ ^10^ pancreatic cancer^11^ and sporadic melanoma.^12^ ^13^ Moreover, highly penetrant germline mutations in the telomere maintenance genes *POT1* and *TERT* have been recently shown in patients with familial melanoma.^14–16^

In our previous LTL meta-GWA, we found that a single-nucleotide polymorphism (SNP) (rs2535913) lies within the gene encoding the DDB1 and CUL4-associated factor 4 (*DCAF4*) had a barely suggestive association with LTL.^4^ In the present study, we present further analysis of rs2535913 through de novo genotyping and in silico look up. As *DDB1* and *CUL4* are engaged in the response to ultraviolet radiation (UVR)^17^ ^18^ and given that the risk for sporadic melanoma is increased with an inherently longer LTL^12^ ^13^ and UVR,^19–21^ we also examined whether rs2535913 is associated with altered expression of DCAF4 in lymphoblastoid cells and sun-exposed skin and whether LTL-associated genes, in general, might also be associated with genes engaged in melanoma pathways.

## Materials and methods

### Cohorts

A detailed description of demographic characteristics of all cohorts included in this study can be found in online supplementary table S1. Additional details related to the discovery and the replication cohorts can be found elsewhere.^2^ ^4^

In brief, for the discovery data, rs2535913 was extracted from the summary results of a large GWA consortium meta-analysis including six cohorts (the Framingham Heart Study, the Family Heart Study, the Cardiovascular Health Study, the Bogalusa Heart Study, the Hypertension Genetic Epidemiology Network Study and TwinsUK). All the cohorts adjusted for the same covariates (age, age^2^, sex, smoking history).^4^ All the samples included in the meta-analysis were of European descent (evidence of non-European ancestry was assessed by principal component analysis comparison with HapMap in each cohort).

LTL measurement was performed by Southern blot analysis of the mean length (expressed in kilobases) of the terminal restriction fragments, generated by the restriction enzymes *Hin*fI and *Rsa*I after verification of DNA integrity.^22^ The de novo genotyping of rs2535913 was conducted on 3037 samples from different cohorts (Israeli Jews from the Jerusalem LRC Longitudinal Study^23^ and Palestinians from the Palestinian-Israeli Jerusalem Risk Factor Study^24^ , Frenchmen from the ADELAHYDE—Nancy study and ERA—France study,^25^ and Danes from a population sample of Danish twins).^26–31^ LTL for these individuals was measured by Southern blots, as in the discovery data set. In the second phase of replication, we performed an in silico look-up of results of LTL-GWAS based on 7795 European descent individuals from four independent cohorts (British Heart Foundation Family Heart Study, Queensland Institute of Medical Research, Brisbane Adolescent Twin Study, United Kingdom Blood Service and an independent sample set from TwinsUK).^2^ Mean LTL of these samples was measured using a qPCR-based technique (ref. 2 and expressed as a ratio of telomere repeat length (T) to a copy number of a single copy gene (S)). A calibrator sample or a standard curve was used to standardise T/S results across plates. LTLs in each cohort were standardised using a Z-score transformation. All the cohorts were also adjusted for age and sex in the main analysis.

### LTL meta-analysis

The meta-analysis for the discovery stages was carried out previously using METAL.^32^ We used GWAMA (V.2.1)^33^ to test the meta-analyses of all the cohorts in which LTL was measured (either by Southern blots or qPCR). To this end, we used the inverse variance weighted method to combine the cohort-specific β-estimates. Because the meta-analysis of the entire data set included samples in which LTL was assessed using two different methods, we used the random-effect inverse variance method implemented in GWAMA. In addition, to test the presence and measure the amount of between-study heterogeneity, we used two different metrics: Cochran's Q statistic^34^ and I^2^.^35^

### Expression analysis

We used the genome-wide expression data from the lymphoblastoid cell lines (LCLs) from the Multiple Tissue Human Expression Resource (MuTHER).^36^ The expression values were derived from a subset of twins from TwinsUK, which were also included in the association analysis. The analysis was performed on rs2535913 and the expression levels of *DCAF4* using MERLIN,^37^ taking into account the family structure. For this analysis, the significance was defined as p<0.05, as only one independent test was performed.

To validate our results in a different data set and to perform tissue-specific (sun-exposed skin from lower leg) expression analysis, we used data from the Genotype-Tissue Expression (GTEx) project online portal (http://www.gtexportal.org).^38^

### Ingenuity pathway analysis

The meta-GWAS data set included in the ingenuity pathway analysis (IPA) Core analysis consisted of 99 773 SNPs that met quality control and had a p value threshold of <0.05.

All SNPs mapping in coding region of a gene or within 2 kb upstream/0.5 kb downstream of it were annotated. Using these criteria, IPA successfully mapped 42 395 of the 99 773 initial SNPs to genes. We compiled and analysed four subsets of genes meeting increasingly stringent p value thresholds in the meta-GWA of LTL (p<0.05: 7362 genes; p<0.01: 2846 genes; p<0.005: 1771 genes; and p<0.001: 526 genes).

We then compared the LTL-associated genes included in the generated subsets with the genes reported by the IPA Global Canonical Pathway (database accessed on September 2014; http://www.ingenuity.com) for melanoma. We finally generated a p value using a 2×2 Fisher's exact test comparing the disease vs non-disease status in the LTL-associated genes and in the reference data set (see online supplementary figure S1). All p values were corrected for multiple testing using the Benjamini–Hochberg method.^39^

## Results and discussion

Meta-GWAS findings in European descendants have already indicated significant associations of LTL with SNPs mapped to loci of *TERC, OBFC1* and *CTC1*—key genes engaged in TL regulation.^2–4^ Of the nine SNPs that displayed suggestive associations with LTL (5×10^−7^<p<5×10^−8^) in our previous meta-GWAS in 9190 European descendants,^4^ seven were mapped to these three gene loci, while the remaining two (rs2535913, p=2×10^−7^; rs2806040, p=2.61×10^−7^) lie within *DCAF4*. Because both *DCAF4* variants were in perfect linkage disequilibrium (r^2^=1) (see online supplementary table S2), we focused our attention on rs2535913 and further examined its association with LTL in eight additional cohorts.

We first de novo genotyped 3037 samples from four independent cohorts in which LTL was measured by Southern blots. We found a borderline significant association (possibly due to the small sample size) of the rs2535913 minor allele (A) with short LTL (β=−0.0343; p=6.13×10^−2^; table 1).

**Table 1:** Association of DCAF4 rs2535913 minor allele with leucocyte telomere length in discovery and replication cohorts

Analysis	N	MAF	β (SE)	SE	p Value	I^2^* (%)	Het p†
Discovery	9190	0.3091	−0.0554‡	0.011	2×10^−7^	18	0.30
Replication 1 (de novo genotyped data set)	3037	0.2743	−0.0343‡	0.018	6.13 ×10^−2^	29	0.24
*Combined (discovery+replication 1)*	12 227	0.2991	−0.0505‡	0.009	2.31×10^−8^	21	0.25
Replication 2 (qPCR data set)	7795	0.3144	−0.0451§	0.017	7.84×10^−3^	0	0.70
*Combined (all)*	20 022	0.3061	−0.0493¶	0.008	6.38×10^−10^	0	0.46

*Heterogeneity index I^2^ by Higgins *et al.*^35^

†Cochran’s heterogeneity statistic’s p value.

‡Effect reported in kb relative to the minor allele.

§Effect reported in (T/S) ratio relative to the minor allele.

¶Effect reported for the inverse-variance random-effect meta-analysis.

The overall meta-analysis of the discovery and de novo genotyped cohorts showed a genome-wide significant p value of 2.31×10^−8^. We then performed an in silico look-up of results of LTL-GWAS based on 7795 individuals from four independent cohorts in which LTL was measured by qPCR.^2^ Results showed an association of the rs2535913 minor allele with shorter LTL (β=−0.0451; p=7.8×10^−3^) (table 1). The combined meta-analysis, based on 20 022 individuals from the 14 independent populations (6 in the discovery data set, 4 de novo genotyped and 4 in silico look-up populations), showed a significant association (β=−0.0493; p=6.4×10^−10^) of rs2535913 with LTL.

Due to the different characteristics of the studies included in the meta-analysis, we tested the between-study heterogeneity using two different metrics (Cochran's Q statistic and I^2^). Both methods did not detect between-study heterogeneity (I^2^=0%; Cochran’s Q p=0.46; table 1 and online supplementary figure S2).

Previous studies,^2–4^ as well as the present research, showcase the large samples required to decipher the genetics of LTL in the general population. Jointly, the few genes, including *DCAF4*, that have been found thus far to be associated at a genome-wide significance level with LTL explain <5% of the inter-individual variation in LTL.^2^

In order to identify potential causal alleles in the coding sequence, we looked for variants in tight linkage disequilibrium with rs2535913 (LD; r^2^>0.9 in 1000 Genomes Project European samples). We identified 15 SNPs (see online supplementary table S2) of which only one (rs2806034) was in the coding region causing a synonymous change (serine to serine) in all the different *DCAF4* transcripts. We also performed a conditional analysis, including rs2535913 as a covariate, to identify potential independent secondary signals at this locus. This analysis did not find any significant evidence for an independent signal (see online supplementary figure S3).

Notably, rs2535913 is located within binding motifs for the chromatin organising factor CTCF and Rad21 (see online supplementary table S2).^40^ Rad21 is a component of the cohesin complex involved in DNA repair, apoptosis and chromosome cohesion during the cell cycle. CTCF and cohesin are both integral components of most human subtelomeric regions and have been implicated in telomere maintenance.^41^

**Figure 1:**
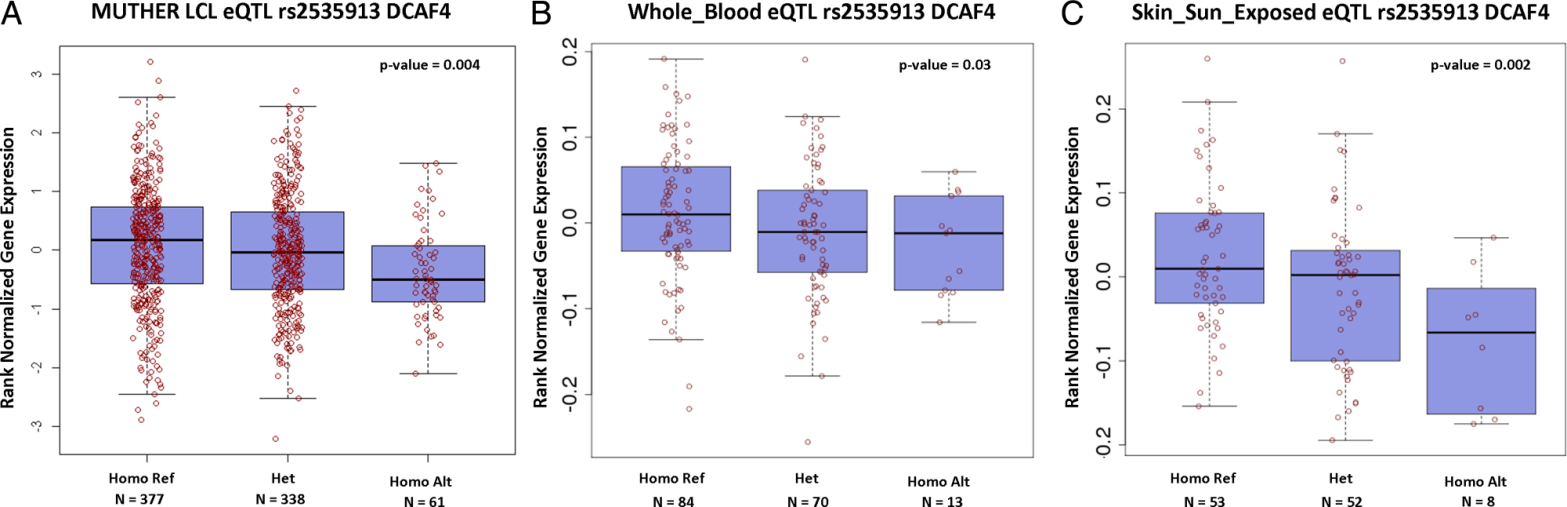
rs2535913-*DCAF4* expression analysis in (A) MuTHER lymphoblastoid cell line (LCL) samples; (B) whole blood and (C) sun-exposed skin tissues using Genotype-Tissue Expression online database. Homo Ref, homozygous GG; Het, heterozygous GA; Homo Alt, homozygous AA.

To explore the potentially functional impact of this intronic SNP on *DCAF4*, we used genome-wide expression data from MuTHER^36^ (http://www.muther.ac.uk/) based on 778 unselected European descendant twins. We first focused our analysis on LCLs. We found that the minor allele (A) of rs2535913 was associated with lower expression of *DCAF4* (β=-0.039, p=4.1×10^−3^) (figure 1A). To validate our results in an independent data set, we used data from the GTEx project online portal (http://www.gtexportal.org).^38^ We found a significant association (p=3×10^−2^) between rs2535913 and DCAF4 expression levels measured in whole blood of 167 individuals (figure 1B).

DCAF4 interacts with DDB1 and CUL4.^18^ This interaction suggests that DCAF4 may be involved in UVR response since DDB1 and DDB2 serve as key detectors of UVR-induced DNA damage and transcription-coupled repair pathways,^17^ ^18^ while cullins are engaged in ubiquitin-dependent protein degradation.^42^ We, therefore, examined rs2535913-DCAF4 expression association specifically in sun-exposed skin (lower leg) tissue included in the GTEx database. Notably, despite the small sample size (n=113), we observed a significant association (p=2×10^−3^) between rs2535913 minor allele and lower DCAF4 expression levels in sun-exposed skin (figure 1C). Thus, the finding of the LTL-*DCAF4* link might be important not only because it expands the repertoire of common SNPs associated with LTL at genome-wide significant level, but also because it may provide a possible link between TL, as expressed in leucocytes, and UVR.

DDB1 modulates the transcription factor E2F1, which, in turn, regulates cell proliferation and telomerase.^43–45^ Thus, cell replication and telomere dynamics might be linked to pathways engaged in UVR-induced DNA damage repair.^46^ In this context, the association of LTL with *DCAF4* might be mediated by telomerase, perhaps via E2F1.^43^ ^44^ ^45^ That is because mutations indicative of UVR damage in the promoter region of *TERT* are common in melanoma tumours (but very rare in the germline) and apparently generate consensus DNA binding sites, which are targets not only of ETS transcription factors^47^ that direct cytoplasmic signals to control gene expression but also E2F1.^48^

We have thus established that *DCAF4* is an LTL-associated gene and that rs2535913 minor allele is associated with decreased *DCAF4* expression in blood and sun-exposed skin, which may suggest *DCAF4* involvement in UVR response. Given that LTL is inherently long in patients with melanoma^12^ ^13^ and the increased risk for this cancer with UVR exposure,^19^ ^20^ ^21^ we sought further links between LTL-associated genes and melanoma in the general population by testing a polygenic model using the results of our large LTL meta-GWA.^4^ This model does not require studying patients with melanoma and is based on the following premise: <10% of melanoma cases are familial.^49^ However, based on research in twins, the heritability of sporadic melanoma is approximately 55%.^50^ It follows that while familial melanoma is caused by highly penetrant and rare germline mutations, sporadic melanoma might result from the additive effect of common genetic variants in the general population, each of which causally contributes a low risk for the disease.^51^ A proof of concept for this premise comes from a recent study in 11 108 melanoma patients and 13 933 controls.^52^ The study developed a genetic risk score for melanoma based on seven LTL-associated genes, which had been derived from LTL-GWA studies in the general population.^2–4^ Similarly, our polygenic approach has been to take advantage of IPA Core analysis to decipher connections of LTL-associated genes in the general population with genes that had been reported to be engaged in melanoma pathways. To this end, we analysed sets of LTL-associated SNPs that met increasingly stringent p value thresholds in our LTL meta-GWA , as described under 'Materials and methods' (see online supplementary table S3). We then compared the number of LTL-associated genes identified by IPA with the total number of genes related to melanoma in the IPA reference set (see online supplementary figure S1). We found that genes included in the melanoma pathway were over-represented with p values ranging from 1.97×10^−169^ (LTL-associated SNPs p value <0.05) to 3.42×10^−24^ (LTL-associated SNPs p value <0.001) in each LTL-associated gene subset (table 2 and see online supplementary figure S1).

**Table 2:** Ingenuity pathway analysis results using increasingly stringent p value thresholds in the LTL meta-GWAS

LTL meta-GWAS threshold	LTL meta-GWAS genes	Genes present in melanoma pathway	p Value
<0.05	7362	3642	1.97×10^−169^
<0.01	2846	1562	1.08×10^−100^
<0.005	1771	999	3.96×10^−70^
<0.001	526	305	3.42×10^−24^

GWAS, genome-wide association studies; LTL, leucocyte telomere length.

LTL-associated genes were also enriched for genes included in pathways of different types of cancers, although at much less significance than that found for melanoma pathways. We observed, for example, that genes included in the colorectal cancer pathway were over-represented among LTL-associated genes with p values ranging from 8.57×10^−62^ (LTL-associated SNPs p value <0.05) to 1.28×10^−6^ (LTL-associated SNPs p value <0.001).

While findings of a longer LTL in sporadic melanoma are fairly consistent across studies, until recently, no consistency had emerged from studies of the relationship between LTL and other cancers.^53^ ^54^ This might be because studies that examined the association of LTL with cancer largely used leucocyte DNA from patients that had already been subjected to chemotherapy, irradiation or both, which probably impacted haematopoiesis and consequently LTL.^53^ ^54^ Moreover, sample sizes of the majority of these studies were often too small, which limited the ability to detect significant effects, particularly when qPCR was used to measure telomere DNA content (due to the large measurement error of this method).^55^ That being said, recent large-scale prospective studies, which include pooled data, now show that inherently long LTL is associated with lung adenoma, as well as the cancers of breast and pancreas.^7–11^ Although hardly applying to all cancers, these findings suggest that an inherently longer LTL might not be unique to patients with melanoma. Cancer is not a single disease, and its causes are multifactorial. Thus, the potential role of telomere biology in carcinogenesis must be contextualised with specific circumstances that depend on the type of cancer, its anatomical location, the age and sex of the individual and his/her overall genetic makeup with respect to a host of heritable and environmental risks.

In conclusion, the core findings of this work are (a) *DCAF4* is a novel gene that contributes to LTL variation in humans and (b) its expression levels are altered in blood and sun-exposed skin; the latter may suggest a potential role in UVR response. We also provide preliminary evidence that genes associated with LTL are enriched among genes engaged in melanoma pathways in the general population. Our model might be useful in testing the role of LTL genetics in other human cancers.

## Supplementary Material

Web supplement
