# Supplementary material for: DCAF4, a novel gene associated with leucocyte telomere length
Source: J Med Genet. 2015 Jan 26;52(3):157–62. doi: 10.1136/jmedgenet-2014-102681 (PMC4345921; doi:10.1136/jmedgenet-2014-102681)
Supplement: Web supplement [file jmedgenet-2014-102681-s1.pdf]

Supplementary Figure 1

A

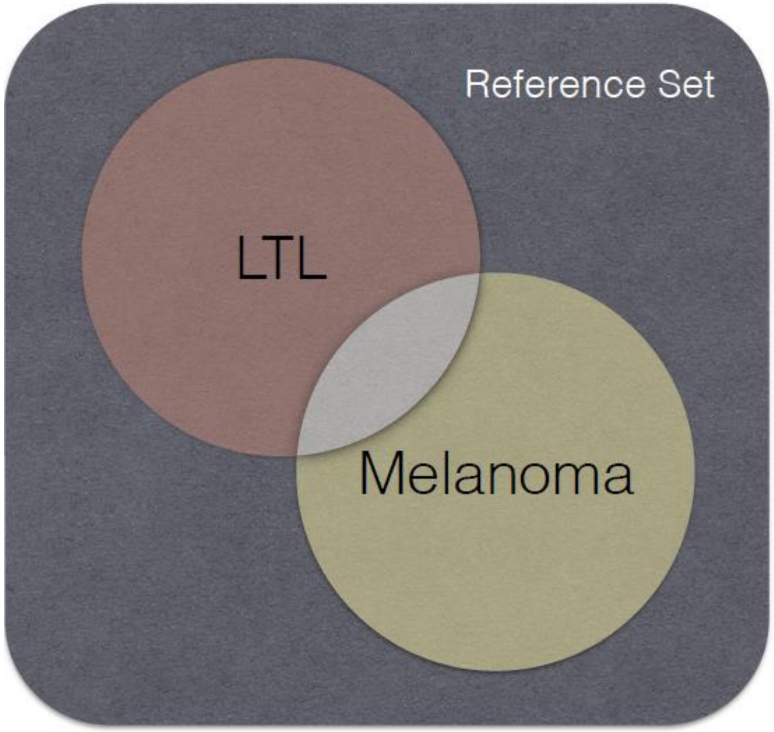

B

	Focus	Non-Focus	Sum
Disease	n_{11}	n_{12}	row_1
Non-Disease	n_{21}	n_{22}	row_2
Sum	col_1	col_2	N

$$p = \frac{row_1!\ row_2!\ col_1!\ col_2!}{N!\ n_{11}!\ n_{12}!\ n_{21}!\ n_{22}!}$$

C

GWAS < 0.05	Focus	Non-Focus	Sum
Disease	3642	4646	8288
Non-Disease	3720	10643	14363
Sum	7362	15289	**22651**

$p = 1.97 \times 10^{-169}$

GWAS < 0.01	Focus	Non-Focus	Sum
Disease	1562	6726	8288
Non-Disease	1284	13079	14363
Sum	2846	19805	**22651**

$p = 1.08 \times 10^{-100}$

GWAS < 0.005	Focus	Non-Focus	Sum
Disease	999	7289	8288
Non-Disease	772	13591	14363
Sum	1771	20880	**22651**

$p = 3.96 \times 10^{-70}$

GWAS p < 0.001	Focus	Non-Focus	Sum
Disease	305	7983	8288
Non-Disease	221	14142	14363
Sum	526	22125	**22651**

$p = 3.42 \times 10^{-24}$

Supplementary Figure 2

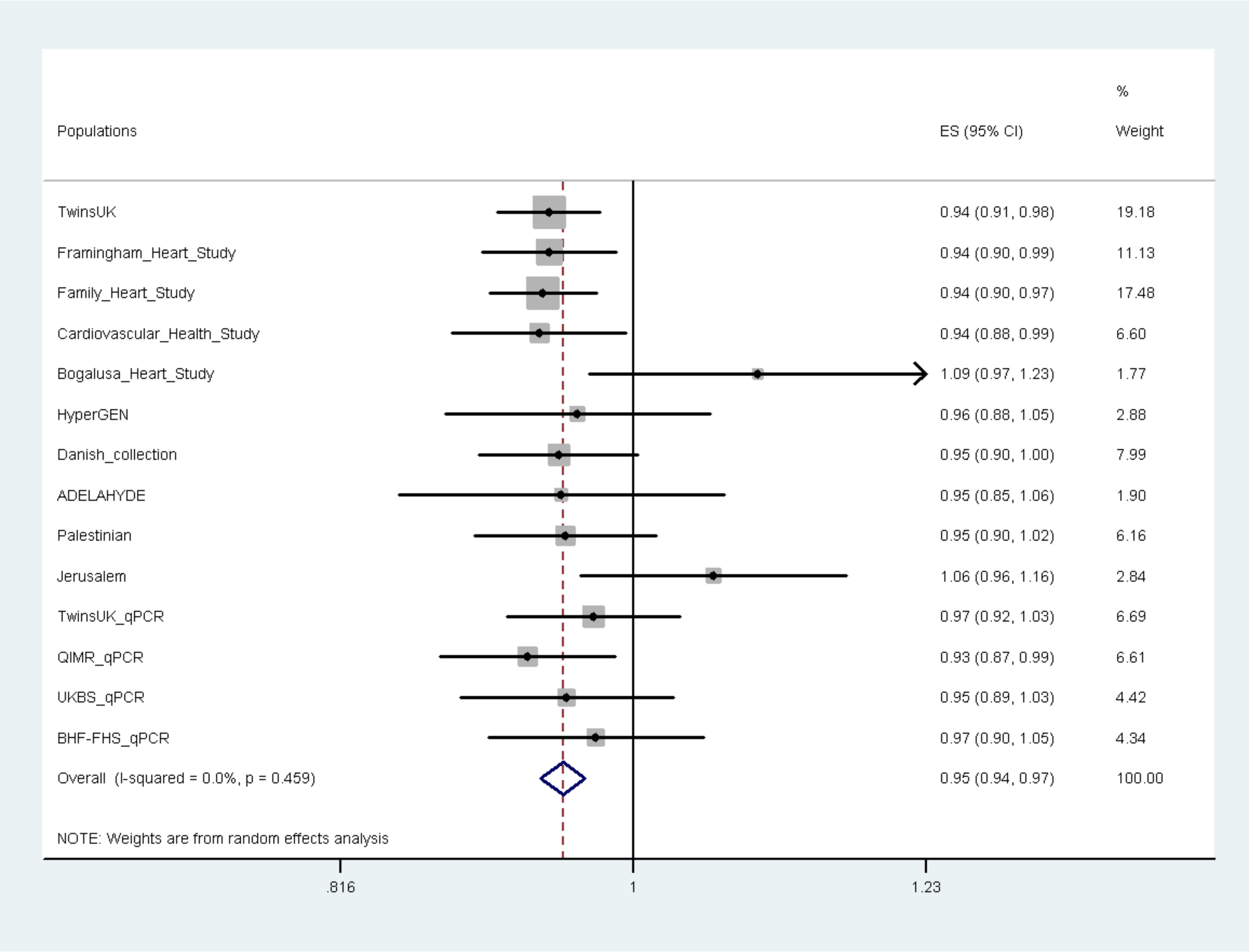

Supplementary Figure 3

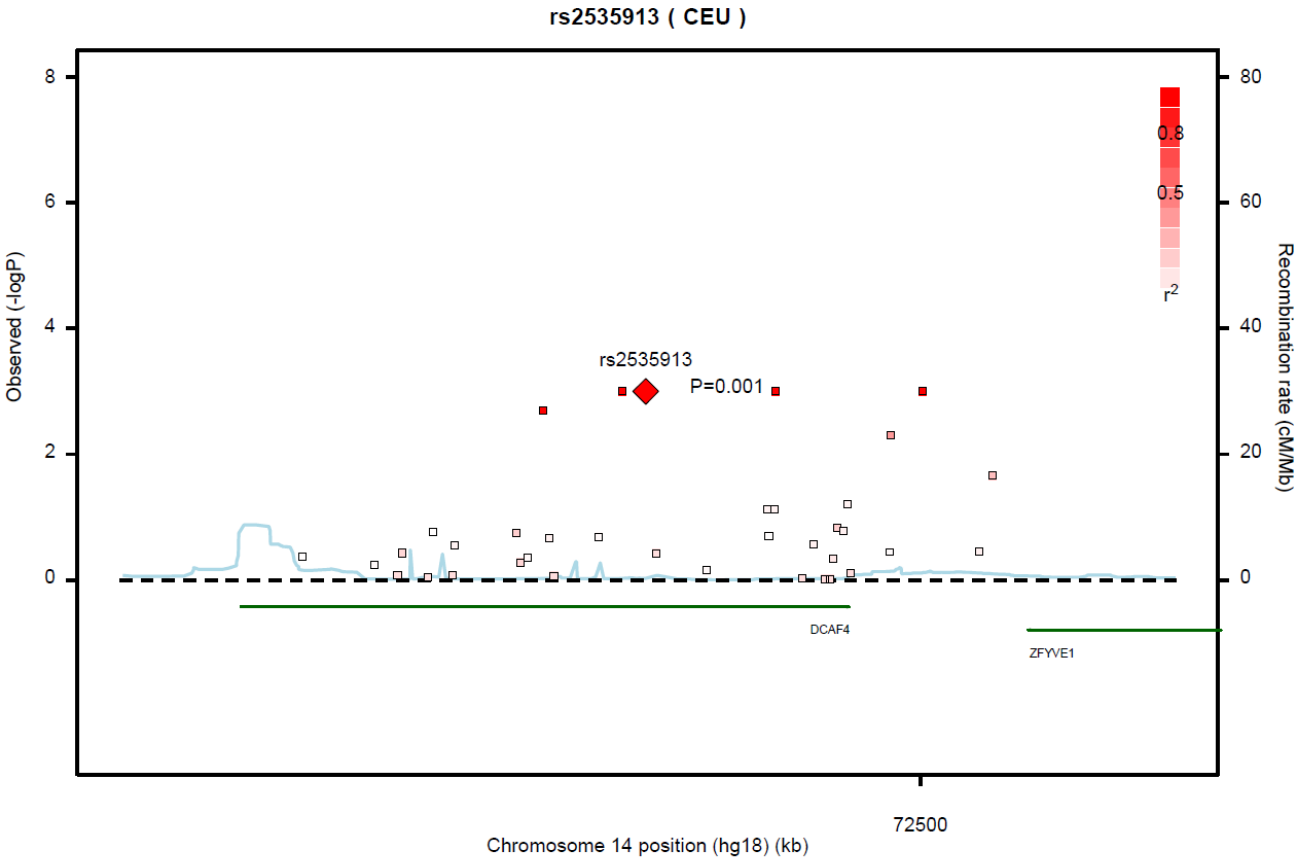

A

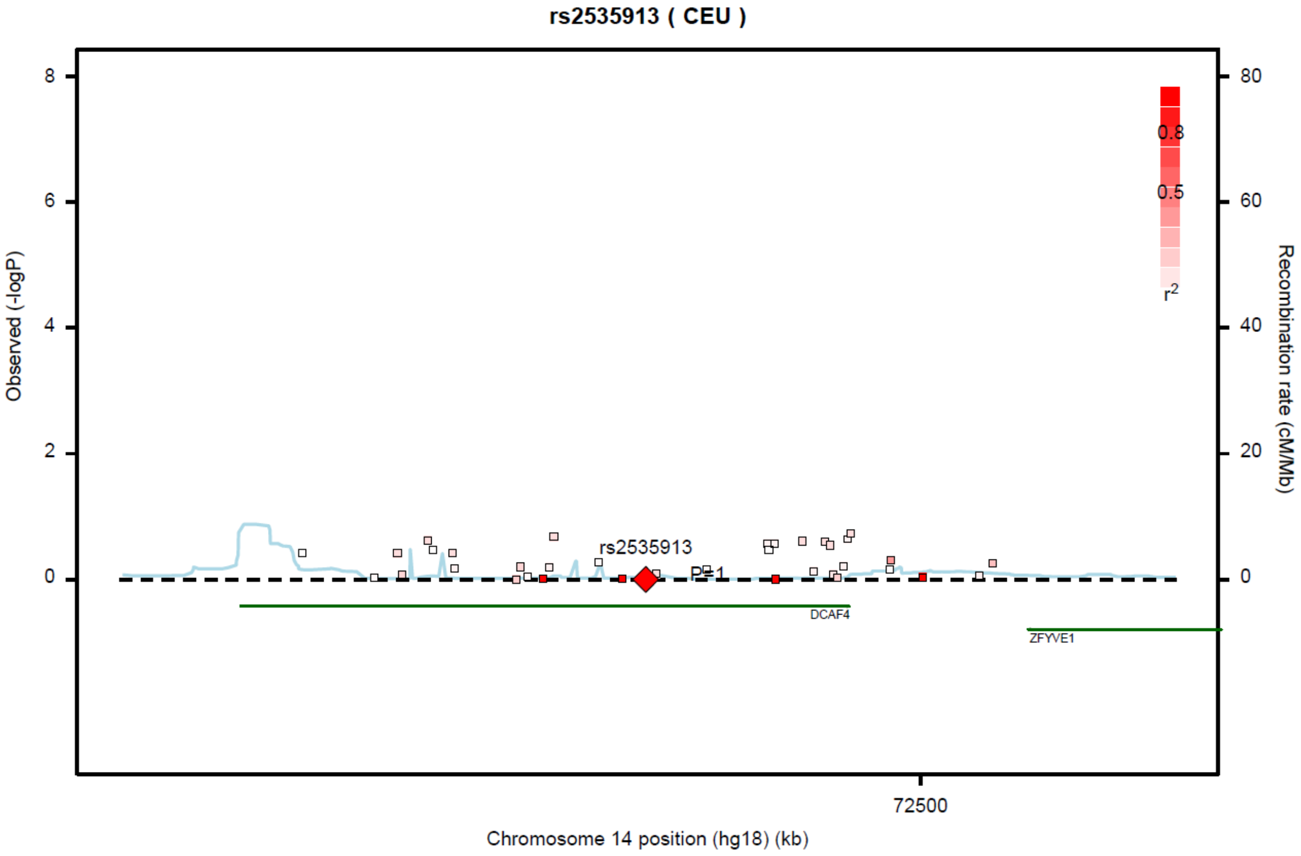

B

Supplementary Figure 1. Summary of the 2x2 grids and method for calculating p-value significance by Fisher exact test**.** (A) General example of 2X2 contingency table used to calculate p-values for the IPA Global Canonical Pathways. (B) Formula (Fisher) to calculate p-value significance of over represented pathways; (C) Four analyzed subsets of LTL associated genes with increasingly stringent p-value thresholds (<0.05,<0.01,<0.005,<0.001) compared with genes included in IPA melanoma pathway. Disease= genes in melanoma pathway; Non-Disease = genes non included in the melanoma pathway; Focus = genes identified by the LTL GWA meta-analysis; Non-Focus = genes included in the IPA reference set; Sum = columns/rows sum.

Supplementary Figure 2. Forest plot displaying an inverse-variance weighted random-effect meta-analysis of the effect of rs2536913 on LTL variation. Effect sizes are plotted with 95% confidence intervals.

Supplementary Figure 3. *DCAF4* locus: A) before and B) after conditioning on rs2536913. The conditional analysis was performed using TwinsUK. After adjusting for rs2535913, all the p-values of the SNPs in linkage disequilibrium were not significant (B). Observed $-\log_{10}(P$ value) are plotted against base-pair position. rs2535913 is represented in red, and the linkage disequilibrium relationship (r^2) with other SNPs in the region are based on the CEU HapMapII samples. Blue peaks represent recombination rates (HapMap 2), and the genes are provided at the bottom.

Supplementary Table 1. Main characteristics for the 12 cohorts included in the analysis

Cohort	N	Mean age (year range)	Women (%)	Mean telomere length (SD)
**Discovery***				
Framingham Heart Study	1146	59 (33 – 86)	51	6.96 (0.58)
Family Heart Study	2,508	57 (30 - 93)	54	6.78 (0.67)
Cardiovascular Health Study	1,061	75 (67 – 95)	62	6.33 (0.61)
Bogalusa Heart Study	333	35 (20 – 48)	42	7.22 (0.70)
HyperGEN	920	50 (18 - 87)	50	6.78 (0.61)
TwinsUK	3,222	48 (18 - 82)	92	6.97 (0.68)
**Replication 1***				
Danish collection	1,376	59.8 (19 - 100)	68	6.33 (0.88)
ADELAHYDE/Nancy/ ERA-France	232	57.1 (25 - 86)	18	6.59 (0.62)
Palestinian	888	51.7 (25 - 77)	47	6.77 (0.61)
Jerusalem LRC	541	43.2 (41 - 46)	34	7.01 (0.64)
**Replication 2****				
TwinsUK***	2513	50.6 (16 – 99)	90	3.82 (0.69)
BHF-FHS[#]	1487	60.8 (32 - 82)	20	1.35 (0.22)
UKBS[##]	1430	43.4 (17 – 69)	52	1.80 (0.50)
QMIR[###]	2371	24.0 (7 - 72)	51	3.49 (0.61)

* Mean telomere length in the Discovery and replication 1 dataset are measured by southern blot and reported in kb

In **Replication 2 mean telomere lengths are measured by qPCR and expressed as T/S ratio (telomere repeat length (T) to copy number of a single copy gene (S))

*** TwinsUK samples included in the replication stage are independent from the discovery dataset.

British Heart Foundation Family Heart Study

Queensland Institute of Medical Research Brisbane Adolescent Twin Study

United Kingdom Blood Service

Supplementary Table 2. SNPs in linkage disequilibrium ($r^2 < 0.9$) with rs2535913. Linkage disequilibrium (LD) values and minor allele frequencies (MAF) for each SNPs were calculated from the 1000 Genomes Phase 1. The motif changes were predicted by HaploReg V2.

Marker	Chr	Position (BUILD37)	LD (r^2)	Ref. Allele	Alt. Allele	MAF (EUR)	MAF (AFR)	MAF (AMR)	MAF (ASN)	Motifs changed	dbSNP func. annot.
rs72732496	14	73399850	0.98	C	T	0.33	0.2	0.17	0.13	4 altered motifs	intronic
rs11626756	14	73401157	0.97	C	T	0.33	0.2	0.17	0.13	9 altered motifs	intronic
rs28393070	14	73404290	0.99	G	A	0.33	0.2	0.17	0.13	LUN-1	intronic
rs2302587	14	73404795	0.99	G	A	0.33	0.2	0.17	0.13		intronic
rs2333012	14	73409613	0.99	G	A	0.33	0.21	0.17	0.13	Maf,NF-E2	intronic
rs2535914	14	73410621	1	C	T	0.33	0.2	0.17	0.13	CTCF,Maf	intronic
rs2246976	14	73412739	1	C	T	0.33	0.2	0.17	0.13	Ets	intronic
rs2243443	14	73413965	1	G	C	0.33	0.2	0.17	0.13	HNF4,SEF-1	intronic
rs2806041	14	73414895	1	G	T	0.33	0.25	0.19	0.19	8 altered motifs	intronic
rs2806040	14	73415065	1	T	C	0.33	0.2	0.17	0.13	5 altered motifs	intronic
**rs2535913**	**14**	**73415233**	**1**	**G**	**A**	**0.33**	**0.2**	**0.17**	**0.13**	**CTCF,Rad21**	**intronic**
rs2806038	14	73417863	1	A	G	0.33	0.2	0.17	0.14	5 altered motifs	intronic
rs2535912	14	73418200	1	T	C	0.33	0.2	0.17	0.13	4 altered motifs	intronic
rs2535910	14	73420642	0.94	G	A	0.33	0.2	0.17	0.13	AP-1,Foxa	intronic
rs2806034	14	73422350	0.94	T	G	0.33	0.21	0.18	0.14	NRSF,RAR,Sin3Ak-20	synonymous

Supplementary Table 3. List of all 3642 LTL-associated genes (p value threshold of $p<0.05$) with melanoma function annotation described by the IPA Global Canonical Pathway (database accessed on September 2014). The reported p values for each gene are from previous LTL GWA meta-analysis.

Gene	LTL GWAS Significance	Gene	LTL GWAS Significance	Gene	LTL GWAS Significance	Gene	LTL GWAS Significance
A2ML1	<0.05	DPCD	<0.05	MAP3K10	<0.05	SEC16B	<0.005
A4GALT	<0.05	DPEP2	<0.05	MAP3K13	<0.05	SEC24B	<0.01
AACS	<0.05	DPF3	<0.001	MAP3K19	<0.005	SEC31A	<0.01
AADACL3	<0.05	DPP10	<0.01	MAP3K2	<0.05	SEC63	<0.005
AADAT	<0.05	DPP4	<0.005	MAP3K5	<0.005	SECISBP2L	<0.01
AAK1	<0.05	DPP6	<0.005	MAP3K7	<0.005	SEL1L3	<0.005
ABAT	<0.001	DPP8	<0.05	MAP3K9	<0.05	SEMA3C	<0.005
ABCA1	<0.05	DPY19L2	<0.01	MAP4K3	<0.005	SEMA3D	<0.05
ABCA12	<0.005	DPYD	<0.05	MAP4K4	<0.001	SEMA3E	<0.05
ABCA13	<0.001	DPYS	<0.05	MAP7	<0.005	SEMA4D	<0.05
ABCA3	<0.01	DRC1	<0.005	MAP9	<0.05	SEMA6D	<0.005
ABCA4	<0.05	DRD2	<0.005	MAPK1	<0.05	SENP2	<0.01
ABCA6	<0.01	DRD3	<0.05	MAPK10	<0.05	SENP5	<0.05
ABCA8	<0.05	DROSHA	<0.05	MAPK4	<0.005	SENP7	<0.05
ABCB1	<0.01	DSC3	<0.005	MAPK8IP1	<0.05	SEPHS1	<0.05
ABCB4	<0.05	DSCAM	<0.001	MARCH10	<0.05	SEPP1	<0.01
ABCB5	<0.05	DSCAML1	<0.01	MARCO	<0.05	SEPT14	<0.05
ABCC1	<0.001	DSE	<0.05	MARK1	<0.05	SERGEF	<0.01
ABCC11	<0.05	DSG1	<0.05	MARS2	<0.05	SERINC3	<0.05
ABCC3	<0.05	DSP	<0.05	MASP1	<0.01	SERPINA12	<0.05
ABCC4	<0.005	DST	<0.005	MAST3	<0.05	SERPINA3	<0.01
ABCC8	<0.005	DSTN	<0.05	MAST4	<0.05	SERPINA4	<0.05
ABCC9	<0.005	DSTYK	<0.05	MATN2	<0.05	SERPINA5	<0.005
ABCD2	<0.05	DTNA	<0.005	MB21D2	<0.05	SERPINA9	<0.05
ABCD3	<0.005	DTNBP1	<0.05	MBNL1	<0.05	SERPINB11	<0.05
ABCE1	<0.05	DUOX1	<0.05	MBOAT4	<0.05	SERPINB7	<0.001
ABCG8	<0.01	DUOX2	<0.005	MCAM	<0.05	SERPINH1	<0.005
ABL1	<0.05	DUSP22	<0.05	MCC	<0.001	SERPINI2	<0.05
ABLIM1	<0.05	DUXA	<0.05	MCCC1	<0.005	SERTM1	<0.05
ABLIM2	<0.01	DVL3	<0.05	MCHR2	<0.05	SESN2	<0.05
ABLIM3	<0.001	DYM	<0.05	MCM6	<0.001	SETBP1	<0.01
ABR	<0.005	DYNC1I1	<0.05	MCMBP	<0.01	SETD2	<0.005
ABTB2	<0.05	DYNC1LI2	<0.05	MCOLN3	<0.05	SETD4	<0.05
ACACA	<0.05	DYNC2H1	<0.005	MCPH1	<0.001	SETD5	<0.05
ACACB	<0.05	DYRK1A	<0.005	MCTP2	<0.05	SETX	<0.05
ACAN	<0.005	DYRK4	<0.05	MDN1	<0.05	SEZ6L	<0.05
ACCS	<0.005	DYSF	<0.001	MECOM	<0.005	SF3B1	<0.05
ACCSL	<0.005	DZANK1	<0.05	MED12L	<0.05	SF3B2	<0.005
ACER2	<0.005	DZIP1	<0.05	MED13L	<0.05	SFMBT1	<0.001
ACIN1	<0.05	E2F8	<0.05	MED15	<0.05	SFMBT2	<0.001
ACMSD	<0.001	EBF1	<0.005	MED23	<0.05	SGCG	<0.005
ACOT11	<0.05	EBF2	<0.05	MED27	<0.05	SGCZ	<0.005
ACOXL	<0.01	EBF3	<0.05	MED31	<0.05	SGIP1	<0.05

ACPT	<0.05	ECE1	<0.05	MED9	<0.05	SGMS1	<0.001
ACSL3	<0.05	ECT2L	<0.001	MEGF11	<0.005	SGOL2	<0.01
ACSL5	<0.05	EDAR	<0.05	MEGF6	<0.05	SGPP2	<0.05
ACSM2A	<0.05	EDEM3	<0.05	MEGF8	<0.05	SGSM1	<0.01
ACSM2B	<0.005	EDIL3	<0.001	MEI1	<0.05	SGSM2	<0.005
ACSS1	<0.005	EDNRB	<0.01	MEIS2	<0.05	SH2B1	<0.05
ACTA2	<0.05	EEA1	<0.05	MEPE	<0.01	SH2D3A	<0.05
ACTN1	<0.005	EEF1A1	<0.05	MERTK	<0.005	SH2D4A	<0.005
ACTR3B	<0.005	EEF2K	<0.05	METTL11B	<0.05	SH2D4B	<0.05
ACTRT2	<0.05	EEPD1	<0.05	METTL17	<0.05	SH2D7	<0.01
ACVR1C	<0.05	EFCAB13	<0.05	MFHAS1	<0.05	SH3BP4	<0.05
ADA	<0.05	EFCAB5	<0.005	MFN2	<0.001	SH3BP5	<0.05
ADAD2	<0.05	EFCAB6	<0.005	MFNG	<0.05	SH3D19	<0.005
ADAM12	<0.001	EFEMP1	<0.005	MFSD4	<0.05	SH3GL2	<0.001
ADAM18	<0.005	EGF	<0.05	MFSD5	<0.05	SH3GL3	<0.05
ADAM19	<0.05	EGFLAM	<0.05	MFSD9	<0.05	SH3PXD2A	<0.001
ADAM2	<0.005	EGFR	<0.005	MGA	<0.01	SH3PXD2B	<0.05
ADAM22	<0.05	EHD1	<0.005	MGAM	<0.05	SH3RF2	<0.05
ADAM23	<0.05	EHD2	<0.005	MGAT3	<0.05	SH3TC1	<0.05
ADAM28	<0.005	EHD3	<0.05	MGAT4C	<0.001	SH3TC2	<0.05
ADAM29	<0.05	EHD4	<0.05	MGAT5	<0.01	SHANK1	<0.001
ADAM7	<0.01	EIF2AK1	<0.05	MGAT5B	<0.005	SHANK2	<0.001
ADAM9	<0.01	EIF2AK2	<0.01	MGLL	<0.01	SHB	<0.005
ADAMTS12	<0.01	EIF4G3	<0.05	MGMT	<0.005	SHC3	<0.001
ADAMTS14	<0.005	ELAVL2	<0.05	MICAL2	<0.05	SHC4	<0.001
ADAMTS17	<0.01	ELAVL4	<0.01	MICAL3	<0.01	SHISA6	<0.001
ADAMTS18	<0.005	ELF2	<0.05	MICALCL	<0.005	SHOC2	<0.05
ADAMTS19	<0.001	ELF5	<0.05	MICALL1	<0.005	SHROOM3	<0.005
ADAMTS2	<0.005	ELMO1	<0.005	MICU3	<0.05	SI	<0.05
ADAMTS20	<0.05	ELMOD1	<0.01	MIEF1	<0.05	SIDT1	<0.05
ADAMTS3	<0.05	ELMSAN1	<0.05	MIOS	<0.001	SIGIRR	<0.05
ADAMTS4	<0.05	ELTD1	<0.05	MIPEP	<0.005	SIK3	<0.005
ADAMTS5	<0.005	EMB	<0.05	MITF	<0.05	SIN3B	<0.05
ADAMTS6	<0.01	EMID1	<0.05	MKI67	<0.05	SIPA1L1	<0.05
ADAMTS7	<0.05	EMILIN2	<0.05	MKL1	<0.05	SIPA1L2	<0.005
ADAMTS8	<0.05	EML1	<0.001	MKNK1	<0.05	SIPA1L3	<0.001
ADAMTS9	<0.05	EML4	<0.05	MKX	<0.05	SIRPB1	<0.001
ADAMTSL1	<0.005	EML5	<0.01	MLH1	<0.05	SIRPG	<0.05
ADAMTSL3	<0.005	EML6	<0.005	MLIP	<0.005	SIRT4	<0.05
ADAMTSL4	<0.01	EMR3	<0.05	MLKL	<0.005	SKA1	<0.05
ADARB1	<0.05	ENAH	<0.05	MLLT3	<0.005	SKAP1	<0.01
ADARB2	<0.001	ENDOU	<0.05	MLYCD	<0.05	SKI	<0.05
ADCK2	<0.05	ENDOV	<0.05	MME	<0.005	SKIV2L2	<0.05
ADCY1	<0.01	ENOX1	<0.01	MMP14	<0.05	SKP2	<0.05
ADCY10	<0.01	ENPEP	<0.01	MMP16	<0.005	SLAIN1	<0.05
ADCY2	<0.005	ENPP3	<0.05	MMP19	<0.05	SLAMF6	<0.05
ADCY7	<0.05	ENTHD1	<0.05	MMP27	<0.05	SLAMF8	<0.01
ADCY8	<0.005	ENTPD1	<0.05	MN1	<0.01	SLC10A2	<0.05
ADCY9	<0.001	ENTPD4	<0.01	MNDA	<0.05	SLC10A7	<0.05
ADCYAP1R1	<0.05	EP300	<0.05	MOCOS	<0.005	SLC12A5	<0.05

ADD2	<0.001	EP400	<0.01	MOV10L1	<0.05	SLC12A6	<0.05
ADD3	<0.001	EPAS1	<0.05	MPDZ	<0.005	SLC13A1	<0.05
ADORA3	<0.05	EPB41L4A	<0.01	MPHOSPH9	<0.001	SLC13A3	<0.05
ADRA1A	<0.005	EPB41L4B	<0.01	MPL	<0.05	SLC13A5	<0.05
ADRA1B	<0.05	EPC2	<0.05	MPO	<0.05	SLC14A2	<0.005
AEBP2	<0.01	EPG5	<0.01	MPP4	<0.05	SLC15A1	<0.05
AFF1	<0.05	EPHA10	<0.05	MPP6	<0.05	SLC16A12	<0.05
AFF3	<0.005	EPHA3	<0.05	MPP7	<0.05	SLC16A7	<0.05
AFMID	<0.05	EPHA4	<0.05	MPPED2	<0.05	SLC16A9	<0.05
AGA	<0.05	EPHA5	<0.005	MR1	<0.001	SLC17A5	<0.005
AGAP1	<0.01	EPHA6	<0.05	MRAS	<0.05	SLC17A8	<0.005
AGBL1	<0.005	EPHA7	<0.005	MREG	<0.05	SLC17A9	<0.005
AGBL2	<0.05	EPHA8	<0.05	MRGPRX2	<0.05	SLC18A1	<0.05
AGBL4	<0.01	EPHB1	<0.005	MROH5	<0.05	SLC19A2	<0.05
AGL	<0.005	EPHB2	<0.05	MROH8	<0.001	SLC19A3	<0.05
AGMO	<0.01	EPHB4	<0.05	MROH9	<0.01	SLC1A6	<0.005
AGO2	<0.05	EPHB6	<0.01	MRPL30	<0.05	SLC22A15	<0.05
AGPAT3	<0.05	EPN1	<0.05	MRVI1	<0.05	SLC22A16	<0.01
AGPAT4	<0.05	EPN2	<0.01	MS4A1	<0.005	SLC22A3	<0.01
AGPAT9	<0.01	EPRS	<0.05	MS4A14	<0.05	SLC22A5	<0.005
AGTPBP1	<0.05	EPS15L1	<0.05	MS4A6A	<0.05	SLC22A6	<0.01
AGXT2	<0.05	EPS8	<0.005	MSGN1	<0.05	SLC22A8	<0.05
AHCTF1	<0.001	EPYC	<0.05	MSH4	<0.05	SLC24A3	<0.005
AHCY	<0.05	ERBB4	<0.005	MSI1	<0.01	SLC25A12	<0.05
AHNAK	<0.01	ERC2	<0.005	MSRA	<0.05	SLC25A21	<0.01
AIF1	<0.005	ERCC3	<0.05	MST1	<0.05	SLC25A37	<0.05
AIM1	<0.05	ERCC6	<0.05	MTCH1	<0.005	SLC25A47	<0.05
AIM1L	<0.05	ERG	<0.005	MTCH2	<0.05	SLC25A48	<0.05
AJAP1	<0.001	ERGIC1	<0.05	MTCL1	<0.01	SLC26A11	<0.05
AK7	<0.05	ERICH3	<0.01	MTDH	<0.05	SLC26A3	<0.05
AK9	<0.05	ERICH6	<0.05	MTFR2	<0.01	SLC26A4	<0.01
AKAP13	<0.01	ERMP1	<0.05	MTHFD1L	<0.01	SLC26A7	<0.05
AKAP3	<0.05	ERN2	<0.05	MTIF3	<0.05	SLC26A9	<0.01
AKAP6	<0.01	ESPL1	<0.05	MTMR3	<0.05	SLC27A2	<0.05
AKR1C3	<0.001	ESRRB	<0.05	MTMR7	<0.05	SLC27A3	<0.01
AKR1C4	<0.05	ESRRG	<0.005	MTO1	<0.05	SLC27A6	<0.05
AKR1D1	<0.05	ESYT2	<0.001	MTR	<0.001	SLC28A1	<0.05
AKT3	<0.05	ETAA1	<0.05	MTRF1	<0.05	SLC28A2	<0.05
ALDH18A1	<0.05	ETFB	<0.05	MTTP	<0.05	SLC28A3	<0.01
ALDH1A2	<0.01	ETS1	<0.01	MTUS1	<0.01	SLC2A12	<0.05
ALDH1L2	<0.005	ETV3L	<0.05	MTUS2	<0.05	SLC2A5	<0.05
ALDH2	<0.01	ETV5	<0.05	MUC16	<0.05	SLC2A9	<0.05
ALDH8A1	<0.05	ETV6	<0.001	MUC3A	<0.05	SLC30A8	<0.01
ALG1	<0.005	EVA1C	<0.05	MUC5B	<0.01	SLC30A9	<0.005
ALG10	<0.05	EVC	<0.01	MVB12B	<0.01	SLC33A1	<0.05
ALK	<0.005	EVC2	<0.05	MX2	<0.05	SLC35D2	<0.05
ALKBH3	<0.01	EVL	<0.01	MYCBP2	<0.005	SLC35E3	<0.05
ALOX5	<0.05	EVPL	<0.01	MYCBPAP	<0.01	SLC35F3	<0.01
ALOXE3	<0.005	EVX1	<0.05	MYH10	<0.005	SLC35F4	<0.005
ALPK1	<0.01	EXOC4	<0.05	MYH11	<0.01	SLC36A1	<0.05

ALPK2	<0.01	EXPH5	<0.05	MYH14	<0.05	SLC38A1	<0.005
ALPL	<0.005	EXT1	<0.005	MYH15	<0.05	SLC39A12	<0.05
ALS2	<0.005	EYA1	<0.05	MYH3	<0.001	SLC39A4	<0.05
ALS2CL	<0.05	EYA2	<0.01	MYH6	<0.05	SLC40A1	<0.05
AMBN	<0.05	EYA4	<0.005	MYH9	<0.005	SLC41A2	<0.05
AMPD3	<0.05	EYS	<0.001	MYL10	<0.005	SLC44A4	<0.001
AMPH	<0.005	EZH2	<0.05	MYLK	<0.05	SLC44A5	<0.01
ANAPC1	<0.01	F11	<0.05	MYLK2	<0.05	SLC45A1	<0.01
ANGPT1	<0.01	F13A1	<0.005	MYNN	<0.001	SLC46A3	<0.05
ANGPT4	<0.05	F3	<0.05	MYO15A	<0.05	SLC4A10	<0.05
ANGPTL5	<0.01	FA2H	<0.05	MYO16	<0.001	SLC4A11	<0.005
ANK1	<0.05	FABP12	<0.01	MYO18B	<0.005	SLC4A4	<0.05
ANK2	<0.001	FABP2	<0.01	MYO1E	<0.005	SLC4A5	<0.005
ANK3	<0.005	FABP6	<0.001	MYO1G	<0.05	SLC4A7	<0.005
ANKFN1	<0.05	FADS2	<0.05	MYO3A	<0.05	SLC4A8	<0.05
ANKMY1	<0.05	FAM102A	<0.05	MYO3B	<0.005	SLC5A1	<0.05
ANKRD11	<0.05	FAM107A	<0.005	MYO5B	<0.05	SLC5A7	<0.05
ANKRD17	<0.05	FAM107B	<0.01	MYO5C	<0.005	SLC5A8	<0.005
ANKRD24	<0.005	FAM114A2	<0.05	MYO7A	<0.05	SLC5A9	<0.05
ANKRD26	<0.05	FAM120A	<0.05	MYOCD	<0.01	SLC6A1	<0.05
ANKRD27	<0.001	FAM120B	<0.05	MYOF	<0.05	SLC6A11	<0.005
ANKRD28	<0.05	FAM129A	<0.05	MYOM1	<0.05	SLC6A13	<0.05
ANKRD29	<0.01	FAM135B	<0.01	MYOM2	<0.05	SLC6A18	<0.005
ANKRD30A	<0.05	FAM13A	<0.01	MYOM3	<0.05	SLC6A19	<0.01
ANKRD46	<0.05	FAM13B	<0.05	MYPN	<0.05	SLC6A2	<0.005
ANKS1A	<0.01	FAM13C	<0.001	MYRIP	<0.005	SLC6A20	<0.005
ANKS1B	<0.005	FAM149A	<0.05	MYT1L	<0.05	SLC6A4	<0.005
ANO2	<0.005	FAM155A	<0.005	N4BP2	<0.05	SLC6A6	<0.01
ANO3	<0.005	FAM161A	<0.05	NAALADL2	<0.005	SLC7A13	<0.05
ANO4	<0.005	FAM161B	<0.05	NALCN	<0.01	SLC7A2	<0.05
ANO7	<0.05	FAM162B	<0.05	NANOS3	<0.05	SLC7A7	<0.05
ANXA1	<0.05	FAM168A	<0.05	NAP1L1	<0.05	SLC7A9	<0.005
ANXA10	<0.05	FAM171A1	<0.005	NARS2	<0.01	SLC8A2	<0.05
ANXA2	<0.05	FAM171B	<0.05	NAT10	<0.05	SLC8A3	<0.05
AOAH	<0.005	FAM179A	<0.05	NAV2	<0.005	SLC9A2	<0.05
AOX1	<0.01	FAM179B	<0.005	NAV3	<0.01	SLC9A3	<0.05
AP1B1	<0.05	FAM184A	<0.01	NBAS	<0.005	SLC9A9	<0.005
AP1S3	<0.005	FAM189A2	<0.05	NBEA	<0.005	SLC9C1	<0.05
AP2A1	<0.05	FAM193A	<0.05	NBEAL2	<0.05	SLC9C2	<0.005
AP3S1	<0.05	FAM19A2	<0.05	NBN	<0.01	SLCO1A2	<0.05
AP4S1	<0.05	FAM19A4	<0.05	NCALD	<0.05	SLCO1B7	<0.05
APBA1	<0.05	FAM208B	<0.05	NCAM1	<0.05	SLCO1C1	<0.01
APBA2	<0.05	FAM20A	<0.005	NCAM2	<0.001	SLCO2B1	<0.005
APBB1IP	<0.05	FAM212B	<0.05	NCAN	<0.05	SLCO3A1	<0.05
APC2	<0.05	FAM46C	<0.01	NCAPG2	<0.005	SLCO4C1	<0.05
APCDD1L	<0.05	FAM49A	<0.001	NCAPH	<0.05	SLCO5A1	<0.05
APEH	<0.01	FAM53A	<0.05	NCAPH2	<0.05	SLCO6A1	<0.05
APLNR	<0.001	FAM60A	<0.01	NCBP1	<0.05	SLFN14	<0.005
APLP2	<0.05	FAM71B	<0.05	NCKAP1L	<0.01	SLIT1	<0.001
APOA2	<0.05	FAM71D	<0.05	NCKAP5	<0.001	SLIT2	<0.005

APOB	<0.05	FAM71F1	<0.001	NCOA1	<0.01	SLIT3	<0.005
APOL3	<0.05	FAM73B	<0.01	NCOA2	<0.05	SLTM	<0.05
APP	<0.05	FAM83B	<0.01	NCOA5	<0.05	SMAD6	<0.05
AQP1	<0.05	FAM83E	<0.05	NCOA6	<0.05	SMAD9	<0.05
AQPEP	<0.001	FAM83F	<0.05	NCOR2	<0.005	SMAP2	<0.05
ARAP2	<0.001	FAM98A	<0.05	NDNF	<0.05	SMARCA2	<0.005
ARF3	<0.05	FAM98C	<0.05	NDRG4	<0.05	SMARCA4	<0.05
ARFGAP3	<0.05	FAR1	<0.05	NDST1	<0.001	SMARCAD1	<0.05
ARFGEF1	<0.05	FARP1	<0.05	NDST3	<0.05	SMARCC1	<0.01
ARFGEF2	<0.001	FARP2	<0.005	NDST4	<0.01	SMARCD3	<0.05
ARFIP1	<0.05	FARSB	<0.05	NDUFS2	<0.01	SMC2	<0.01
ARFRP1	<0.01	FASN	<0.05	NEB	<0.005	SMC3	<0.001
ARHGAP10	<0.01	FASTKD2	<0.05	NEBL	<0.05	SMC4	<0.005
ARHGAP12	<0.01	FAT1	<0.05	NEDD4	<0.05	SMG7	<0.05
ARHGAP15	<0.05	FAT2	<0.05	NEGR1	<0.001	SMIM7	<0.05
ARHGAP18	<0.05	FAT3	<0.001	NEIL2	<0.01	SMOC1	<0.01
ARHGAP22	<0.005	FAT4	<0.05	NEK10	<0.05	SMOC2	<0.01
ARHGAP24	<0.005	FBLIM1	<0.005	NEK11	<0.05	SMPD3	<0.05
ARHGAP26	<0.001	FBLN1	<0.005	NEK6	<0.05	SMURF1	<0.01
ARHGAP27	<0.05	FBLN2	<0.01	NEK7	<0.01	SMURF2	<0.05
ARHGAP28	<0.05	FBLN5	<0.05	NELFA	<0.05	SMYD1	<0.05
ARHGAP29	<0.05	FBN2	<0.05	NELFE	<0.001	SMYD2	<0.05
ARHGAP32	<0.05	FBXO18	<0.05	NELL1	<0.001	SNAP25	<0.005
ARHGAP44	<0.05	FBXO22	<0.05	NELL2	<0.05	SNAP91	<0.05
ARHGEF10	<0.01	FBXO3	<0.05	NEMF	<0.01	SNED1	<0.005
ARHGEF10L	<0.05	FBXO31	<0.05	NET1	<0.01	SNRPA	<0.05
ARHGEF11	<0.005	FBXO40	<0.01	NETO1	<0.01	SNTB1	<0.001
ARHGEF12	<0.05	FBXW7	<0.005	NF2	<0.05	SNTG2	<0.01
ARHGEF15	<0.01	FCER1A	<0.01	NFASC	<0.005	SNX14	<0.05
ARHGEF16	<0.05	FCGR2B	<0.05	NFATC1	<0.05	SNX17	<0.01
ARHGEF18	<0.05	FCHSD1	<0.05	NFATC2	<0.05	SNX29	<0.005
ARHGEF26	<0.005	FCRLB	<0.001	NFE2L1	<0.05	SOAT1	<0.05
ARHGEF28	<0.05	FECH	<0.05	NFIA	<0.001	SOCS5	<0.01
ARHGEF4	<0.01	FER1L6	<0.05	NFIC	<0.05	SOGA1	<0.05
ARHGEF7	<0.001	FERMT1	<0.05	NFKB1	<0.05	SORBS1	<0.001
ARID1B	<0.001	FGD3	<0.01	NFXL1	<0.05	SORBS2	<0.05
ARID2	<0.05	FGD4	<0.05	NGF	<0.01	SORCS1	<0.05
ARL14	<0.01	FGD5	<0.005	NGFR	<0.05	SORCS3	<0.05
ARMC3	<0.05	FGD6	<0.005	NHEJ1	<0.05	SORL1	<0.05
ARMC4	<0.01	FGF12	<0.05	NID1	<0.01	SOS2	<0.005
ARNT2	<0.05	FGF14	<0.05	NID2	<0.005	SOSTDC1	<0.005
ARNTL	<0.05	FGF2	<0.05	NIM1K	<0.05	SOX10	<0.01
ARPP21	<0.05	FGFR1	<0.05	NIN	<0.05	SOX6	<0.005
ARRB2	<0.05	FGFR2	<0.05	NINJ2	<0.05	SP110	<0.005
ARRDC4	<0.05	FHAD1	<0.005	NIPAL1	<0.05	SP140	<0.01
ARSJ	<0.01	FHDC1	<0.01	NIPBL	<0.01	SP140L	<0.01
ARVCF	<0.005	FHIT	<0.005	NISCH	<0.05	SP4	<0.01
ASAH2	<0.05	FHOD3	<0.001	NLGN1	<0.001	SPACA7	<0.05
ASB13	<0.05	FIBCD1	<0.05	NLRC3	<0.05	SPAG16	<0.05
ASB14	<0.05	FIGLA	<0.005	NLRC5	<0.05	SPAG17	<0.001

ASB4	<0.05	FIP1L1	<0.05	NLRP1	<0.05	SPAG9	<0.05
ASCC3	<0.01	FKTN	<0.05	NLRP11	<0.05	SPAM1	<0.005
ASIC1	<0.05	FLNB	<0.05	NLRP12	<0.01	SPARCL1	<0.05
ASNS	<0.05	FLT1	<0.05	NLRP3	<0.01	SPATA13	<0.005
ASTN1	<0.05	FLT3	<0.05	NLRP5	<0.05	SPATA16	<0.05
ASTN2	<0.005	FLT4	<0.05	NLRP7	<0.005	SPATA5	<0.01
ASXL1	<0.05	FMN1	<0.005	NLRP9	<0.05	SPATA5L1	<0.005
ASXL2	<0.01	FMN2	<0.01	NLRX1	<0.05	SPATA7	<0.005
ASXL3	<0.005	FMO1	<0.05	NME8	<0.05	SPATC1L	<0.05
ATF2	<0.05	FMO2	<0.05	NMT1	<0.05	SPEF2	<0.01
ATF6	<0.05	FN1	<0.001	NMT2	<0.05	SPEG	<0.05
ATF7	<0.05	FNBP4	<0.01	NMUR2	<0.05	SPEN	<0.05
ATF7IP	<0.05	FNDC1	<0.05	NOA1	<0.05	SPERT	<0.05
ATF7IP2	<0.05	FNDC3A	<0.05	NOD1	<0.01	SPHKAP	<0.05
ATG5	<0.05	FNDC3B	<0.005	NOD2	<0.05	SPI1	<0.05
ATHL1	<0.01	FNIP1	<0.05	NODAL	<0.001	SPIC	<0.005
ATIC	<0.05	FOLH1	<0.01	NOL10	<0.005	SPINK5	<0.05
ATL1	<0.05	FOLH1B	<0.001	NOL4	<0.001	SPIRE1	<0.05
ATL2	<0.005	FOLR4	<0.05	NOL9	<0.005	SPNS3	<0.05
ATM	<0.05	FOXK1	<0.005	NOM1	<0.05	SPOCK1	<0.001
ATP10A	<0.05	FOXN1	<0.05	NOP2	<0.05	SPOCK3	<0.01
ATP10B	<0.01	FOXN3	<0.005	NOS1	<0.01	SPP1	<0.05
ATP10D	<0.01	FOXO3	<0.05	NOS1AP	<0.01	SPRED1	<0.05
ATP11A	<0.05	FOXP1	<0.005	NOS2	<0.01	SPRED2	<0.05
ATP13A2	<0.05	FOXP2	<0.05	NOTCH1	<0.05	SPRED3	<0.01
ATP13A4	<0.05	FPR1	<0.05	NOTCH2	<0.05	SPTA1	<0.05
ATP1A2	<0.05	FPR2	<0.05	NOTCH4	<0.05	SPTB	<0.05
ATP1A4	<0.005	FPR3	<0.05	NOX3	<0.05	SPTBN1	<0.01
ATP1B1	<0.005	FRAS1	<0.005	NOX4	<0.001	SPTBN4	<0.05
ATP2B2	<0.005	FREM1	<0.005	NPAS2	<0.01	SPTBN5	<0.05
ATP2B4	<0.05	FREM2	<0.001	NPAS3	<0.001	SPTLC3	<0.005
ATP2C2	<0.001	FRK	<0.05	NPAT	<0.001	SPTSSB	<0.05
ATP6V0A2	<0.05	FRMD3	<0.005	NPC1	<0.01	SPZ1	<0.005
ATP6V0D2	<0.05	FRMD4A	<0.005	NPEPPS	<0.05	SRBD1	<0.05
ATP6V1B1	<0.05	FRMD5	<0.05	NPL	<0.05	SRC	<0.05
ATP6V1E2	<0.05	FRMPD1	<0.05	NPM1	<0.01	SREBF2	<0.05
ATP8A1	<0.01	FRMPD2	<0.01	NPNT	<0.01	SRGAP1	<0.01
ATP8A2	<0.01	FRY	<0.05	NPTXR	<0.05	SRGAP3	<0.005
ATP8B3	<0.05	FRYL	<0.05	NPY2R	<0.01	SRI	<0.05
ATP8B4	<0.05	FSHR	<0.01	NR1I2	<0.05	SRL	<0.05
ATP9A	<0.05	FSIP1	<0.05	NR1I3	<0.05	SRRM4	<0.05
ATR	<0.01	FSIP2	<0.05	NR2C2	<0.05	SRSF3	<0.05
ATRNL1	<0.005	FSTL1	<0.05	NR2E1	<0.005	SSB	<0.05
ATXN1	<0.05	FSTL4	<0.005	NR3C2	<0.05	SSBP2	<0.05
ATXN10	<0.01	FSTL5	<0.001	NRAP	<0.001	SSBP3	<0.05
ATXN2	<0.05	FTCD	<0.05	NRCAM	<0.01	SSH2	<0.05
AURKB	<0.005	FUT8	<0.05	NREP	<0.01	SSH3	<0.05
AXDND1	<0.05	FUT9	<0.005	NRG1	<0.001	SSTR2	<0.05
AXL	<0.05	FYB	<0.005	NRG2	<0.05	SSTR5	<0.05
AZIN1	<0.005	FZD2	<0.05	NRG3	<0.005	ST14	<0.005

B3GALNT1	<0.05	G6PC	<0.05	NRIP1	<0.05	ST18	<0.05
B3GALT1	<0.05	GAA	<0.01	NRP1	<0.01	ST6GAL2	<0.01
B3GALT5	<0.01	GAB1	<0.05	NRP2	<0.05	ST6GALNAC3	<0.005
B4GALNT2	<0.05	GABBR2	<0.005	NRXN1	<0.001	ST6GALNAC5	<0.05
B4GALNT3	<0.05	GABRB1	<0.001	NRXN2	<0.05	ST7	<0.05
B4GALT1	<0.05	GABRB2	<0.01	NRXN3	<0.001	ST8SIA1	<0.05
BAAT	<0.05	GABRB3	<0.01	NSD1	<0.001	ST8SIA2	<0.005
BACH2	<0.05	GAK	<0.05	NSL1	<0.005	ST8SIA4	<0.05
BAG6	<0.005	GALNT1	<0.05	NSUN3	<0.01	ST8SIA5	<0.05
BAI3	<0.01	GALNT10	<0.001	NT5DC1	<0.005	STAB2	<0.05
BAIAP3	<0.05	GALNT13	<0.001	NT5E	<0.05	STAC	<0.05
BANF2	<0.05	GALNT14	<0.05	NTF3	<0.005	STAP1	<0.05
BANK1	<0.01	GALNT15	<0.05	NTM	<0.005	STARD13	<0.005
BARX2	<0.005	GALNT16	<0.05	NTN4	<0.05	STARD3NL	<0.005
BAZ1B	<0.05	GALNT5	<0.01	NTNG1	<0.05	STARD6	<0.001
BAZ2B	<0.001	GALNT8	<0.05	NTRK3	<0.001	STAT4	<0.05
BBS12	<0.05	GALNTL5	<0.01	NTSR1	<0.05	STEAP1B	<0.01
BBS4	<0.05	GALNTL6	<0.001	NUAK1	<0.05	STEAP3	<0.05
BBX	<0.05	GANAB	<0.05	NUCKS1	<0.05	STEAP4	<0.05
BCAS1	<0.05	GAP43	<0.01	NUDCD1	<0.05	STIL	<0.005
BCAS3	<0.05	GAREM	<0.05	NUDT1	<0.05	STIM2	<0.05
BCAT2	<0.05	GARS	<0.05	NUF2	<0.05	STK10	<0.05
BCHE	<0.05	GAS2	<0.05	NUFIP2	<0.05	STK17A	<0.05
BCL11A	<0.05	GAS2L3	<0.01	NUGGC	<0.005	STK19	<0.01
BCL11B	<0.01	GBA	<0.05	NUP107	<0.005	STK3	<0.005
BCL2	<0.01	GBA3	<0.05	NUP153	<0.05	STK31	<0.005
BCL9	<0.005	GBE1	<0.005	NUP160	<0.005	STK32B	<0.05
BCR	<0.05	GBP1	<0.05	NUP188	<0.05	STK39	<0.001
BDH2	<0.01	GBP4	<0.005	NUP210	<0.01	STK40	<0.05
BDKRB2	<0.05	GBP5	<0.01	NUP214	<0.05	STOML3	<0.01
BDP1	<0.05	GBP6	<0.01	NUP98	<0.005	STON1-GTF2A1L	<0.005
BEND4	<0.01	GBP7	<0.05	NUTM1	<0.05	STON2	<0.05
BEST3	<0.05	GC	<0.05	NVL	<0.05	STOX1	<0.05
BFSP1	<0.05	GCH1	<0.05	NWD1	<0.05	STOX2	<0.05
BID	<0.05	GCK	<0.05	NXN	<0.01	STPG2	<0.05
BIN1	<0.05	GCKR	<0.05	NXPE4	<0.05	STRBP	<0.01
BIRC5	<0.05	GCLC	<0.05	NXPH1	<0.005	STRN	<0.05
BLK	<0.05	GCN1L1	<0.005	NXPH2	<0.001	STX8	<0.005
BLM	<0.05	GDA	<0.005	NXPH3	<0.05	STXBP5L	<0.05
BLZF1	<0.05	GDF6	<0.05	NYAP2	<0.01	STYK1	<0.005
BMP5	<0.01	GDF9	<0.05	OBSCN	<0.05	SUCLG2	<0.05
BMPER	<0.005	GDNF	<0.01	OGDHL	<0.05	SUCO	<0.005
BMPR1B	<0.01	GDPD4	<0.05	OLFM3	<0.005	SULF1	<0.001
BMPR2	<0.005	GDPD5	<0.05	OLFM4	<0.005	SULF2	<0.005
BNC2	<0.005	GEN1	<0.005	OLFML3	<0.05	SULT1C3	<0.005
BPIFB6	<0.05	GFAP	<0.05	ONECUT2	<0.001	SULT2A1	<0.01
BPIFC	<0.01	GFRA1	<0.01	OPCML	<0.001	SULT2B1	<0.05
BRCA2	<0.05	GFRA3	<0.05	OPRD1	<0.05	SUN1	<0.05
BRD3	<0.01	GFRAL	<0.05	OPRK1	<0.01	SUN5	<0.05
BRD7	<0.05	GGT6	<0.05	OR10A3	<0.005	SUPT5H	<0.05

BRD9	<0.05	GHR	<0.001	OR10A5	<0.05	SUPT7L	<0.05
BRE	<0.05	GHRH	<0.001	OR10AG1	<0.05	SUPV3L1	<0.005
BRINP1	<0.001	GHRHR	<0.005	OR10G2	<0.05	SV2A	<0.05
BRINP2	<0.05	GHSR	<0.05	OR10G4	<0.01	SV2B	<0.01
BRINP3	<0.05	GID4	<0.05	OR10G9	<0.05	SV2C	<0.01
BSN	<0.01	GIGYF1	<0.05	OR10S1	<0.01	SVEP1	<0.005
BTAF1	<0.05	GIMAP2	<0.05	OR11L1	<0.05	SVIL	<0.005
BTBD11	<0.005	GIP	<0.05	OR12D3	<0.05	SVOP	<0.01
BTBD8	<0.05	GJB6	<0.05	OR13A1	<0.05	SVOPL	<0.05
BTBD9	<0.05	GLB1	<0.005	OR13F1	<0.05	SWAP70	<0.05
BTD	<0.05	GLDC	<0.05	OR1G1	<0.05	SYCP2L	<0.05
BTN2A1	<0.05	GLDN	<0.05	OR1L8	<0.005	SYK	<0.05
BTN2A2	<0.05	GLG1	<0.001	OR1N2	<0.05	SYN3	<0.005
BTN3A1	<0.05	GLI2	<0.05	OR2C1	<0.05	SYNDIG1	<0.001
BTN3A3	<0.05	GLIS1	<0.05	OR4C15	<0.05	SYNE1	<0.01
BTNL9	<0.005	GLIS3	<0.005	OR51F1	<0.05	SYNE2	<0.005
BUB1B	<0.05	GLMN	<0.05	OR51G1	<0.05	SYNJ1	<0.05
BZRAP1	<0.05	GLOD4	<0.05	OR52B4	<0.05	SYNJ2	<0.05
BZW2	<0.005	GLP1R	<0.05	OR52H1	<0.05	SYNM	<0.05
C10orf128	<0.005	GLRA3	<0.001	OR5AS1	<0.01	SYNPO2	<0.05
C10orf129	<0.05	GLRB	<0.005	OR5H1	<0.05	SYNPO2L	<0.05
C10orf53	<0.05	GLT8D2	<0.05	OR5H14	<0.05	SYT1	<0.01
C10orf90	<0.01	GLYATL1	<0.05	OR5H15	<0.05	SYT10	<0.01
C11orf57	<0.05	GLYATL3	<0.05	OR5I1	<0.005	SYT16	<0.05
C11orf63	<0.05	GMEB2	<0.05	OR5P3	<0.05	SYT3	<0.05
C12orf29	<0.01	GMPR	<0.05	OR5T1	<0.01	SYT7	<0.001
C12orf40	<0.05	GNA11	<0.01	OR5T2	<0.05	SYT9	<0.005
C12orf42	<0.05	GNAI1	<0.05	OR5W2	<0.05	SZT2	<0.05
C12orf50	<0.05	GNAI2	<0.05	OR6C1	<0.05	T	<0.05
C12orf55	<0.005	GNAL	<0.05	OR6C2	<0.05	TAAR5	<0.05
C12orf56	<0.05	GNAQ	<0.01	OR6C4	<0.05	TACC2	<0.05
C14orf159	<0.05	GNAS	<0.05	OR6C75	<0.05	TACR1	<0.01
C14orf166	<0.05	GNB4	<0.01	OR7D4	<0.005	TACR3	<0.05
C14orf37	<0.05	GNPTG	<0.005	OR8H2	<0.05	TAF1C	<0.05
C14orf39	<0.01	GOLIM4	<0.05	OR8H3	<0.05	TAF2	<0.01
C15orf52	<0.05	GOSR2	<0.01	OR8I2	<0.05	TAF4B	<0.01
C16orf62	<0.05	GOT2	<0.05	OR9G4	<0.005	TAF8	<0.01
C16orf80	<0.05	GPALPP1	<0.05	ORC1	<0.01	TAL1	<0.05
C17orf104	<0.05	GPATCH4	<0.05	ORC5	<0.05	TANC1	<0.01
C17orf70	<0.05	GPC5	<0.01	OSBP2	<0.01	TANGO2	<0.05
C17orf80	<0.05	GPCPD1	<0.01	OSBPL10	<0.005	TAOK3	<0.005
C17orf85	<0.01	GPD1L	<0.05	OSBPL1A	<0.001	TAP1	<0.05
C18orf54	<0.005	GPHN	<0.01	OSBPL2	<0.01	TAP2	<0.005
C18orf8	<0.05	GPI	<0.05	OSBPL3	<0.005	TARBP1	<0.05
C19orf47	<0.05	GPLD1	<0.05	OSBPL5	<0.05	TAS1R2	<0.01
C1QC	<0.05	GPM6A	<0.05	OSGIN2	<0.05	TAS2R7	<0.05
C1RL	<0.05	GPR115	<0.05	OSMR	<0.01	TATDN2	<0.05
C1orf105	<0.05	GPR116	<0.05	OSTF1	<0.05	TBC1D1	<0.01
C1orf127	<0.05	GPR123	<0.05	OTOF	<0.05	TBC1D13	<0.005
C1orf226	<0.005	GPR128	<0.01	OTOG	<0.01	TBC1D14	<0.05

C1orf54	<0.005	GPR133	<0.01	OTUD7A	<0.05	TBC1D16	<0.05
C1orf87	<0.05	GPR137B	<0.05	OVCH2	<0.01	TBC1D30	<0.05
C1orf94	<0.01	GPR139	<0.05	OXCT1	<0.005	TBC1D31	<0.05
C2	<0.005	GPR156	<0.05	OXR1	<0.005	TBC1D4	<0.005
C20orf26	<0.05	GPR158	<0.05	P2RX7	<0.05	TBCD	<0.05
C22orf46	<0.01	GPR161	<0.05	P2RY2	<0.05	TBCE	<0.05
C2CD2	<0.005	GPR176	<0.005	PACS1	<0.05	TBCEL	<0.05
C2orf42	<0.05	GPR26	<0.01	PACSIN1	<0.05	TBL1XR1	<0.005
C2orf43	<0.001	GPR39	<0.05	PAFAH1B1	<0.05	TBX15	<0.05
C2orf83	<0.05	GPR55	<0.05	PAH	<0.01	TBX18	<0.05
C3orf20	<0.05	GPR56	<0.05	PAK6	<0.005	TBX3	<0.01
C3orf67	<0.05	GPR63	<0.001	PAK7	<0.005	TBXAS1	<0.05
C4orf22	<0.005	GPR65	<0.05	PALD1	<0.05	TCEA2	<0.05
C5	<0.01	GPR83	<0.05	PALLD	<0.001	TCF4	<0.005
C6orf10	<0.05	GPR98	<0.05	PALM	<0.05	TCF7L1	<0.05
C6orf106	<0.05	GPRC5D	<0.05	PALM2-AKAP2	<0.05	TCHP	<0.05
C6orf165	<0.05	GPRC6A	<0.05	PANK3	<0.05	TCN2	<0.05
C7orf60	<0.05	GRAMD1B	<0.05	PAOX	<0.05	TCP10L	<0.05
C8orf34	<0.005	GRAMD1C	<0.05	PAPD4	<0.05	TCP11	<0.005
C8orf74	<0.05	GRAP2	<0.05	PAPL	<0.05	TCP11L1	<0.05
C9orf129	<0.05	GREB1	<0.05	PAPLN	<0.005	TCP11L2	<0.05
C9orf170	<0.005	GRHL1	<0.01	PAPPA	<0.01	TDRD1	<0.05
C9orf41	<0.05	GRHL2	<0.05	PAPPA2	<0.05	TDRD12	<0.001
CA10	<0.005	GRIA1	<0.005	PAPSS1	<0.05	TDRD7	<0.05
CA7	<0.05	GRIA2	<0.005	PARD3B	<0.005	TDRD9	<0.05
CA8	<0.05	GRIA4	<0.01	PARK2	<0.005	TEAD4	<0.05
CA9	<0.05	GRID1	<0.001	PARN	<0.01	TEC	<0.001
CAAP1	<0.05	GRID2	<0.001	PARP14	<0.005	TECPR2	<0.01
CABIN1	<0.001	GRIK1	<0.01	PARP4	<0.005	TECRL	<0.005
CABP4	<0.05	GRIK2	<0.001	PARVB	<0.005	TECTA	<0.005
CABP5	<0.05	GRIK3	<0.01	PATL1	<0.005	TEK	<0.05
CACNA1A	<0.05	GRIK4	<0.001	PAX2	<0.01	TENM2	<0.005
CACNA1B	<0.005	GRIN2A	<0.01	PAX3	<0.005	TENM3	<0.005
CACNA1C	<0.001	GRIN2B	<0.005	PAX6	<0.05	TENM4	<0.001
CACNA1D	<0.005	GRIN2D	<0.005	PAX7	<0.01	TEP1	<0.05
CACNA1E	<0.05	GRIN3A	<0.005	PBLD	<0.05	TERT	<0.001
CACNA1G	<0.005	GRIP1	<0.05	PBX1	<0.005	TESPA1	<0.05
CACNA1H	<0.005	GRIP2	<0.01	PC	<0.05	TET1	<0.05
CACNA1I	<0.01	GRK4	<0.01	PCBP3	<0.005	TEX15	<0.005
CACNA1S	<0.01	GRK5	<0.001	PCDH10	<0.005	TEX33	<0.05
CACNA2D1	<0.001	GRM1	<0.01	PCDH15	<0.005	TF	<0.05
CACNA2D3	<0.001	GRM3	<0.05	PCDH17	<0.05	TFAP2A	<0.05
CACNB2	<0.001	GRM4	<0.005	PCDH9	<0.001	TFEB	<0.05
CACNG3	<0.005	GRM5	<0.05	PCLO	<0.001	TFEC	<0.05
CACNG4	<0.005	GRM6	<0.05	PCNT	<0.05	TFF2	<0.005
CADM1	<0.01	GRM7	<0.001	PCNXL2	<0.01	TFPI	<0.001
CADM2	<0.01	GRM8	<0.001	PCOLCE2	<0.001	TFR2	<0.05
CADM3	<0.001	GSG1	<0.05	PCSK1	<0.05	TG	<0.01
CADPS	<0.005	GSTA1	<0.05	PCSK5	<0.001	TGFA	<0.05
CADPS2	<0.005	GTDC1	<0.05	PCSK9	<0.05	TGFB2	<0.001

CALB1	<0.05	GTF3C2	<0.05	PDE10A	<0.01	TGFB3	<0.05
CALB2	<0.05	GTPBP4	<0.05	PDE11A	<0.01	TGFBR2	<0.01
CALCR	<0.05	GUCA1C	<0.05	PDE1A	<0.005	TGM3	<0.001
CALD1	<0.05	GUCY1A2	<0.005	PDE1B	<0.001	TGM6	<0.05
CALN1	<0.005	GUCY2C	<0.05	PDE1C	<0.005	THADA	<0.005
CAMK1D	<0.01	GYLTL1B	<0.05	PDE2A	<0.05	THBS4	<0.05
CAMK1G	<0.005	GYPA	<0.05	PDE3A	<0.01	THEMIS	<0.05
CAMK4	<0.005	GYPB	<0.05	PDE4B	<0.05	THRAP3	<0.05
CAMKK1	<0.05	GZF1	<0.005	PDE4D	<0.01	THSD7A	<0.01
CAMKK2	<0.05	H2AFY2	<0.005	PDE6A	<0.05	THSD7B	<0.01
CAMSAP2	<0.05	HABP2	<0.05	PDE6C	<0.05	THUMPD2	<0.01
CAMSAP3	<0.05	HAL	<0.05	PDE7B	<0.005	TIAM2	<0.05
CAMTA1	<0.001	HAO2	<0.05	PDE8A	<0.01	TICRR	<0.005
CAND2	<0.05	HAPLN1	<0.05	PDE8B	<0.05	TIE1	<0.05
CAPN1	<0.05	HBP1	<0.05	PDE9A	<0.005	TIMD4	<0.05
CAPN13	<0.01	HCK	<0.05	PDGFB	<0.05	TIMM17A	<0.01
CAPN14	<0.05	HCN1	<0.01	PDGFC	<0.01	TINAG	<0.005
CAPN2	<0.05	HCRTR2	<0.05	PDGFD	<0.005	TIPARP	<0.05
CARD11	<0.001	HDAC9	<0.001	PDGFRB	<0.005	TJP2	<0.05
CARD6	<0.05	HDLBP	<0.001	PDHA2	<0.005	TLDC1	<0.01
CASC5	<0.05	HEATR4	<0.05	PDHB	<0.05	TLE1	<0.05
CASP10	<0.05	HEATR5A	<0.05	PDILT	<0.05	TLE3	<0.05
CASP14	<0.05	HECA	<0.01	PDK1	<0.001	TLE4	<0.005
CASP8AP2	<0.005	HECTD1	<0.01	PDLIM5	<0.05	TLL1	<0.05
CASQ2	<0.01	HECTD4	<0.01	PDS5A	<0.05	TLL2	<0.001
CASR	<0.05	HECW1	<0.005	PDSS2	<0.005	TLN2	<0.005
CASZ1	<0.05	HECW2	<0.01	PDYN	<0.05	TLR4	<0.01
CATSPER1	<0.01	HEG1	<0.05	PDZD2	<0.005	TM4SF18	<0.001
CATSPERB	<0.001	HELLS	<0.05	PDZRN3	<0.005	TM4SF4	<0.005
CAV1	<0.001	HELZ	<0.05	PDZRN4	<0.05	TM9SF4	<0.01
CBL	<0.005	HEPHL1	<0.05	PEAR1	<0.005	TMC1	<0.005
CBX5	<0.005	HERC1	<0.001	PEBP4	<0.01	TMC2	<0.001
CC2D2A	<0.05	HERC2	<0.05	PENK	<0.05	TMC3	<0.05
CCBE1	<0.05	HERC3	<0.05	PER3	<0.05	TMC5	<0.05
CCDC102B	<0.005	HERC6	<0.001	PES1	<0.05	TMC7	<0.05
CCDC108	<0.05	HHIPL1	<0.05	PEX10	<0.05	TMCC3	<0.05
CCDC114	<0.005	HIF1A	<0.05	PEX13	<0.05	TMCO3	<0.05
CCDC126	<0.05	HIF3A	<0.05	PEX16	<0.01	TMEFF2	<0.01
CCDC129	<0.05	HIPK1	<0.05	PEX26	<0.05	TMEM108	<0.05
CCDC130	<0.05	HIPK2	<0.01	PFKP	<0.001	TMEM132B	<0.005
CCDC14	<0.01	HIPK3	<0.05	PGM2	<0.005	TMEM132C	<0.005
CCDC141	<0.05	HIVEP1	<0.001	PGR	<0.05	TMEM132D	<0.005
CCDC146	<0.005	HIVEP2	<0.05	PHACTR2	<0.005	TMEM132E	<0.01
CCDC147	<0.005	HIVEP3	<0.01	PHC2	<0.05	TMEM135	<0.01
CCDC148	<0.01	HK2	<0.05	PHF20	<0.005	TMEM143	<0.05
CCDC15	<0.005	HKDC1	<0.005	PHF21A	<0.001	TMEM161B	<0.05
CCDC150	<0.05	HLA-C	<0.05	PHGDH	<0.005	TMEM178A	<0.05
CCDC152	<0.05	HLA-DOB	<0.05	PHIP	<0.01	TMEM2	<0.01
CCDC158	<0.05	HLA-DQA2	<0.05	PI16	<0.05	TMEM212	<0.001
CCDC168	<0.01	HLA-DRA	<0.05	PI3	<0.05	TMEM215	<0.01

CCDC170	<0.005	HMCN1	<0.05	PI4KB	<0.05	TMEM217	<0.05
CCDC171	<0.05	HMGCLL1	<0.05	PIAS1	<0.001	TMEM246	<0.05
CCDC172	<0.01	HMGCS2	<0.05	PIBF1	<0.005	TMEM38A	<0.05
CCDC173	<0.01	HNF1A	<0.01	PID1	<0.001	TMEM44	<0.005
CCDC178	<0.001	HNRNPA1	<0.005	PIEZO2	<0.001	TMEM45A	<0.005
CCDC19	<0.05	HNRNPDL	<0.05	PIGG	<0.005	TMEM59L	<0.05
CCDC30	<0.005	HNRNPLL	<0.05	PIGQ	<0.05	TMEM61	<0.05
CCDC33	<0.01	HNRNPR	<0.05	PIGU	<0.005	TMEM63C	<0.005
CCDC37	<0.01	HNRNPUL1	<0.05	PIK3AP1	<0.01	TMEM68	<0.001
CCDC38	<0.005	HOXC9	<0.05	PIK3C2G	<0.05	TMEM74B	<0.05
CCDC60	<0.001	HOXD3	<0.05	PIK3C3	<0.005	TMEM92	<0.05
CCDC62	<0.05	HP1BP3	<0.05	PIK3CA	<0.05	TMEM98	<0.05
CCDC65	<0.05	HPCAL1	<0.05	PIK3R1	<0.05	TMPRSS11A	<0.005
CCDC66	<0.05	HPGDS	<0.05	PIK3R4	<0.05	TMPRSS11D	<0.05
CCDC67	<0.005	HPS5	<0.05	PIK3R5	<0.05	TMPRSS15	<0.005
CCDC73	<0.05	HR	<0.05	PIP5K1C	<0.05	TMPRSS5	<0.01
CCDC81	<0.01	HRASLS5	<0.05	PITPNC1	<0.005	TMPRSS6	<0.005
CCDC83	<0.05	HRH1	<0.005	PITPNM2	<0.001	TMPRSS9	<0.05
CCDC87	<0.05	HRNR	<0.01	PITPNM3	<0.01	TMTC1	<0.01
CCDC88A	<0.05	HS3ST1	<0.05	PITRM1	<0.001	TMTC3	<0.005
CCDC88C	<0.01	HS3ST2	<0.05	PIWIL4	<0.05	TMTC4	<0.05
CCDC91	<0.05	HS3ST4	<0.001	PKD1L2	<0.05	TNC	<0.05
CCDC92	<0.05	HS6ST1	<0.05	PKD2	<0.05	TNFRSF10A	<0.05
CCHCR1	<0.05	HS6ST3	<0.005	PKD2L1	<0.05	TNFRSF11A	<0.01
CCR3	<0.005	HSD17B2	<0.01	PKD2L2	<0.05	TNFRSF19	<0.05
CCSER1	<0.005	HSP90AA1	<0.05	PKHD1	<0.01	TNFRSF1B	<0.005
CCSER2	<0.005	HSPD1	<0.05	PKHD1L1	<0.05	TNFRSF21	<0.05
CD109	<0.01	HTN1	<0.05	PKMYT1	<0.05	TNFRSF4	<0.05
CD180	<0.05	HTN3	<0.05	PKP4	<0.05	TNFSF11	<0.05
CD200	<0.01	HTR1E	<0.05	PLA1A	<0.05	TNFSF14	<0.05
CD200R1L	<0.005	HTR2A	<0.005	PLA2G15	<0.05	TNFSF8	<0.05
CD226	<0.01	HTR3B	<0.005	PLA2G16	<0.05	TNIP1	<0.005
CD274	<0.005	HTR3D	<0.05	PLA2G1B	<0.01	TNKS2	<0.005
CD276	<0.05	HTR4	<0.05	PLA2G2C	<0.005	TNPO1	<0.05
CD300A	<0.05	HTRA1	<0.01	PLA2G4A	<0.05	TNR	<0.005
CD300E	<0.005	HTT	<0.05	PLA2G4C	<0.05	TNRC18	<0.005
CD4	<0.005	HUNK	<0.05	PLA2G5	<0.05	TNRC6A	<0.005
CD44	<0.001	HYDIN	<0.05	PLA2G6	<0.05	TNRC6B	<0.05
CD5	<0.05	IARS	<0.05	PLA2R1	<0.05	TNS1	<0.005
CD59	<0.05	IARS2	<0.01	PLAGL1	<0.001	TNS3	<0.05
CD5L	<0.05	IBSP	<0.05	PLB1	<0.01	TNXB	<0.01
CD82	<0.05	ICA1	<0.001	PLCB1	<0.001	TOMM7	<0.05
CD8B	<0.05	ICK	<0.05	PLCB2	<0.05	TOMM70A	<0.01
CD96	<0.01	IDH1	<0.05	PLCB4	<0.05	TOR3A	<0.05
CDC14A	<0.05	IDO2	<0.05	PLCE1	<0.05	TOX	<0.05
CDC42BPA	<0.005	IFIH1	<0.05	PLCG1	<0.05	TOX2	<0.05
CDC42BPG	<0.05	IFIT5	<0.05	PLCG2	<0.01	TP53BP1	<0.05
CDC42EP4	<0.005	IFNLR1	<0.05	PLCH1	<0.05	TP63	<0.001
CDCP1	<0.05	IFT122	<0.05	PLCL2	<0.005	TP73	<0.005
CDH1	<0.01	IFT140	<0.005	PLCXD2	<0.001	TPCN1	<0.05

CDH11	<0.01	IFT172	<0.01	PLCXD3	<0.01	TPD52	<0.05
CDH13	<0.001	IFT74	<0.05	PLCZ1	<0.05	TPD52L1	<0.05
CDH17	<0.05	IGDCC4	<0.05	PLD1	<0.05	TPGS2	<0.005
CDH18	<0.05	IGF2BP3	<0.05	PLD3	<0.05	TPRG1	<0.05
CDH2	<0.05	IGF2R	<0.05	PLD5	<0.001	TPRX1	<0.05
CDH22	<0.05	IGFN1	<0.01	PLEK	<0.05	TPX2	<0.05
CDH23	<0.05	IGSF10	<0.05	PLEKHA4	<0.05	TRAF2	<0.05
CDH26	<0.005	IGSF11	<0.01	PLEKHA5	<0.01	TRAF3IP1	<0.05
CDH3	<0.05	IGSF21	<0.01	PLEKHA6	<0.005	TRAF3IP3	<0.05
CDH4	<0.005	IGSF3	<0.05	PLEKHA7	<0.05	TRAM2	<0.05
CDH6	<0.01	IGSF5	<0.01	PLEKHG1	<0.001	TRANK1	<0.001
CDH7	<0.05	IGSF9	<0.005	PLEKHG3	<0.001	TRAPPC9	<0.001
CDH8	<0.01	IGSF9B	<0.05	PLEKHG4B	<0.05	TRERF1	<0.05
CDH9	<0.01	IKBKAP	<0.05	PLEKHG5	<0.05	TRHDE	<0.01
CDHR1	<0.005	IKZF1	<0.005	PLEKHH1	<0.005	TRIM10	<0.05
CDHR2	<0.01	IL12A	<0.05	PLEKHH2	<0.05	TRIM15	<0.05
CDHR3	<0.05	IL16	<0.005	PLEKHS1	<0.005	TRIM16	<0.005
CDK13	<0.05	IL18	<0.005	PLG	<0.05	TRIM22	<0.05
CDK14	<0.001	IL1R1	<0.05	PLOD2	<0.05	TRIM26	<0.05
CDK5RAP2	<0.05	IL1RAP	<0.05	PLS1	<0.05	TRIM3	<0.05
CDK7	<0.05	IL1RL1	<0.05	PLXDC2	<0.001	TRIM42	<0.05
CDK8	<0.01	IL1RL2	<0.05	PLXNA1	<0.005	TRIM44	<0.005
CDKN2A	<0.05	IL20RA	<0.05	PLXNA2	<0.001	TRIM55	<0.05
CDO1	<0.05	IL21R	<0.05	PLXNA4	<0.05	TRIM71	<0.05
CDON	<0.05	IL23R	<0.005	PLXNC1	<0.05	TRIM9	<0.01
CDS1	<0.005	IL2RA	<0.05	PMEPA1	<0.05	TRIML1	<0.05
CDYL	<0.01	IL6R	<0.05	PMS2	<0.05	TRIML2	<0.05
CDYL2	<0.005	IL7R	<0.05	PNLDC1	<0.01	TRIO	<0.005
CECR1	<0.01	ILF3	<0.05	PNMA2	<0.05	TRIP11	<0.005
CECR2	<0.005	IMMP2L	<0.005	PNPLA1	<0.05	TRIT1	<0.005
CELA2B	<0.05	IMPG2	<0.05	PNPLA7	<0.05	TRPC3	<0.05
CELF2	<0.001	INADL	<0.05	PNPT1	<0.05	TRPC4	<0.005
CELF4	<0.001	INPP5A	<0.05	PODN	<0.05	TRPC6	<0.05
CELF5	<0.01	INPP5B	<0.005	POGZ	<0.005	TRPC7	<0.05
CELSR1	<0.05	INPP5F	<0.05	POLD1	<0.05	TRPM1	<0.005
CELSR2	<0.005	INPP5K	<0.001	POLE	<0.05	TRPM2	<0.001
CEMIP	<0.01	INSC	<0.005	POLE2	<0.005	TRPM3	<0.005
CENPE	<0.005	INSR	<0.05	POLM	<0.05	TRPM6	<0.005
CENPF	<0.01	INTS10	<0.05	POLQ	<0.05	TRPV5	<0.05
CENPK	<0.005	INTS9	<0.05	POLR1A	<0.05	TRPV6	<0.05
CENPP	<0.05	IP6K3	<0.01	POLR1D	<0.05	TRRAP	<0.05
CENPQ	<0.05	IPCEF1	<0.05	POLR2B	<0.01	TSC1	<0.05
CEP104	<0.05	IPO11	<0.05	POLR3A	<0.05	TSEN2	<0.05
CEP112	<0.005	IPO7	<0.05	POMGNT2	<0.05	TSHR	<0.05
CEP128	<0.05	IPO9	<0.05	POP1	<0.05	TSHZ2	<0.005
CEP135	<0.05	IQCE	<0.05	POPDC3	<0.05	TSHZ3	<0.005
CEP164	<0.01	IQCH	<0.05	POSTN	<0.05	TSNARE1	<0.05
CEP290	<0.05	IQCK	<0.05	POTEA	<0.05	TSPAN18	<0.005
CEP350	<0.01	IQGAP1	<0.05	POU6F2	<0.01	TSPAN2	<0.01
CEP57	<0.05	IQGAP2	<0.05	PPAP2B	<0.05	TSPEAR	<0.005

CEP70	<0.01	IQGAP3	<0.01	PPAPDC1A	<0.01	TTC1	<0.001
CERKL	<0.05	IQSEC3	<0.05	PPARA	<0.05	TTC12	<0.05
CERS3	<0.005	IRAK2	<0.05	PPARG	<0.05	TTC13	<0.05
CERS6	<0.05	IREB2	<0.001	PPARGC1A	<0.05	TTC18	<0.05
CES5A	<0.05	IRF8	<0.05	PPCDC	<0.05	TTC21B	<0.05
CFB	<0.005	IRS1	<0.005	PPFIA2	<0.01	TTC24	<0.05
CFHR4	<0.05	ISG20L2	<0.05	PPFIBP2	<0.005	TTC28	<0.001
CFTR	<0.05	ISX	<0.05	PPL	<0.05	TTC29	<0.05
CGNL1	<0.005	ITFG3	<0.05	PPM1B	<0.05	TTC39C	<0.005
CHAF1B	<0.05	ITGA1	<0.005	PPM1H	<0.005	TTC40	<0.05
CHAT	<0.05	ITGA11	<0.005	PPP1CB	<0.05	TTC7A	<0.005
CHD5	<0.05	ITGA3	<0.01	PPP1R11	<0.05	TTC7B	<0.05
CHD6	<0.05	ITGA4	<0.005	PPP1R16B	<0.01	TTL	<0.05
CHD8	<0.01	ITGA8	<0.05	PPP1R26	<0.05	TTLL1	<0.05
CHD9	<0.001	ITGA9	<0.05	PPP1R36	<0.05	TTLL10	<0.05
CHDH	<0.05	ITGAE	<0.01	PPP1R3A	<0.05	TTLL12	<0.05
CHEK2	<0.05	ITGAL	<0.05	PPP1R42	<0.005	TTLL9	<0.05
CHERP	<0.05	ITGAM	<0.05	PPP2CA	<0.05	TTN	<0.05
CHIT1	<0.05	ITGAV	<0.05	PPP2R2B	<0.05	TTYH2	<0.05
CHL1	<0.05	ITGB3	<0.05	PPP2R2C	<0.01	TUB	<0.05
CHMP2B	<0.01	ITGB5	<0.005	PPP3CA	<0.05	TUBAL3	<0.01
CHRM3	<0.005	ITGB6	<0.05	PPP3CC	<0.05	TUBB4A	<0.05
CHRNA3	<0.05	ITGB8	<0.05	PPP4R1	<0.005	TULP4	<0.01
CHRNA4	<0.01	ITGBL1	<0.01	PPP6C	<0.01	TXNDC2	<0.05
CHRNA5	<0.05	ITIH4	<0.05	PPP6R2	<0.05	TYRP1	<0.05
CHRNB1	<0.05	ITIH5	<0.05	PRAMEF12	<0.05	TYW1	<0.005
CHRNB4	<0.05	ITK	<0.01	PRCP	<0.05	TYW5	<0.05
CHST11	<0.005	ITM2C	<0.05	PRDM1	<0.05	UACA	<0.05
CHST8	<0.01	ITPR1	<0.005	PRDM11	<0.005	UAP1	<0.05
CHST9	<0.05	ITPR2	<0.01	PRDM14	<0.05	UBASH3A	<0.005
CIT	<0.001	ITPR3	<0.01	PRDM16	<0.005	UBE2O	<0.05
CKAP5	<0.05	ITSN1	<0.05	PRDM2	<0.01	UBE3A	<0.05
CLASP2	<0.005	ITSN2	<0.05	PRDM8	<0.01	UBR3	<0.05
CLCA1	<0.01	JAG1	<0.05	PRDM9	<0.05	UBR4	<0.05
CLCN1	<0.05	JAKMIP1	<0.005	PREPL	<0.05	UBR7	<0.05
CLCN6	<0.005	JARID2	<0.05	PREX1	<0.01	UBXN4	<0.01
CLCN7	<0.05	JMJD1C	<0.05	PREX2	<0.005	UBXN7	<0.05
CLCNKA	<0.05	JPH1	<0.05	PRICKLE1	<0.05	UGDH	<0.05
CLDN14	<0.05	JPH2	<0.01	PRICKLE2	<0.005	UGT2B7	<0.005
CLDN18	<0.05	KALRN	<0.001	PRIMA1	<0.005	UGT3A1	<0.05
CLEC12A	<0.05	KANK4	<0.05	PRKAA2	<0.05	UGT3A2	<0.05
CLEC1B	<0.05	KAT2A	<0.05	PRKAG2	<0.01	ULBP1	<0.01
CLEC4F	<0.05	KAT2B	<0.05	PRKAR1A	<0.05	ULK1	<0.05
CLEC5A	<0.005	KAT6B	<0.05	PRKCA	<0.05	ULK2	<0.05
CLIC5	<0.001	KATNAL1	<0.05	PRKCB	<0.01	ULK4	<0.005
CLIC6	<0.05	KATNB1	<0.05	PRKCD	<0.05	UMODL1	<0.05
CLIP1	<0.05	KAZN	<0.005	PRKCG	<0.05	UNC13A	<0.05
CLIP3	<0.05	KBTBD12	<0.05	PRKCH	<0.005	UNC13B	<0.05
CLMP	<0.01	KBTBD7	<0.05	PRKCQ	<0.005	UNC13C	<0.005
CLN6	<0.005	KBTBD8	<0.05	PRKD3	<0.01	UNC13D	<0.05

CLN8	<0.005	KCNA6	<0.001	PRKG1	<0.001	UNC5A	<0.05
CLRN2	<0.05	KCNAB1	<0.01	PRLR	<0.05	UNC5B	<0.005
CLRN3	<0.01	KCNAB2	<0.01	PRMT8	<0.001	UNC5C	<0.005
CLSTN2	<0.005	KCNB1	<0.01	PRNP	<0.05	UNC5D	<0.001
CLTA	<0.05	KCNB2	<0.05	PROM1	<0.005	UNC79	<0.05
CLVS1	<0.005	KCNC2	<0.05	PROM2	<0.01	UNC80	<0.001
CMBL	<0.05	KCNC3	<0.05	PROS1	<0.005	UNC93A	<0.005
CMTR2	<0.05	KCND2	<0.05	PROSER1	<0.005	UPF2	<0.01
CMYA5	<0.05	KCNG4	<0.01	PROX1	<0.05	UQCC1	<0.05
CNBD1	<0.005	KCNH1	<0.05	PRR11	<0.05	URB1	<0.005
CNDP1	<0.05	KCNH5	<0.005	PRR21	<0.001	UROC1	<0.005
CNGA1	<0.05	KCNH7	<0.005	PRR27	<0.05	USH1C	<0.005
CNGA3	<0.01	KCNH8	<0.05	PRR5	<0.05	USH2A	<0.005
CNGB3	<0.005	KCNIP1	<0.005	PRRC2A	<0.001	USO1	<0.05
CNKSR3	<0.01	KCNIP4	<0.005	PRRC2B	<0.05	USP24	<0.05
CNN2	<0.05	KCNJ12	<0.05	PRSS12	<0.05	USP29	<0.05
CNN3	<0.05	KCNJ15	<0.05	PRSS3	<0.005	USP3	<0.001
CNNM1	<0.05	KCNJ16	<0.05	PRSS37	<0.05	USP30	<0.05
CNOT1	<0.05	KCNJ3	<0.05	PRSS38	<0.001	USP31	<0.05
CNOT10	<0.05	KCNJ6	<0.01	PRSS45	<0.05	USP34	<0.001
CNTLN	<0.005	KCNK1	<0.005	PRTFDC1	<0.05	USP43	<0.001
CNTN1	<0.005	KCNK10	<0.005	PRTG	<0.05	USP47	<0.05
CNTN3	<0.001	KCNK13	<0.05	PRUNE2	<0.01	USP53	<0.05
CNTN4	<0.005	KCNK16	<0.001	PSCA	<0.05	UTP20	<0.05
CNTN5	<0.005	KCNK5	<0.05	PSD3	<0.005	UTRN	<0.01
CNTN6	<0.01	KCNK9	<0.01	PSG1	<0.05	VAC14	<0.05
CNTNAP2	<0.001	KCNMA1	<0.005	PSG5	<0.05	VANGL2	<0.05
CNTNAP4	<0.05	KCNN1	<0.05	PSMA1	<0.05	VASH2	<0.05
CNTNAP5	<0.001	KCNN3	<0.001	PSMA8	<0.005	VAT1	<0.05
CNTRL	<0.05	KCNQ1	<0.05	PSME4	<0.001	VAV1	<0.05
COBL	<0.01	KCNQ3	<0.005	PTBP3	<0.05	VAV2	<0.01
COBLL1	<0.05	KCNQ4	<0.01	PTCD2	<0.01	VAV3	<0.001
COG3	<0.05	KCNQ5	<0.001	PTCH1	<0.05	VCAM1	<0.05
COG5	<0.01	KCNS3	<0.05	PTCHD3	<0.005	VCAN	<0.001
COG6	<0.005	KCNT2	<0.005	PTCHD4	<0.05	VCL	<0.05
COL11A1	<0.05	KCTD16	<0.001	PTEN	<0.05	VCPKMT	<0.05
COL11A2	<0.005	KCTD19	<0.05	PTGER3	<0.005	VDR	<0.05
COL12A1	<0.005	KCTD3	<0.005	PTGFRN	<0.05	VEGFC	<0.05
COL13A1	<0.05	KCTD8	<0.05	PTGIS	<0.05	VEZT	<0.005
COL14A1	<0.05	KDM2B	<0.05	PTK2B	<0.01	VIPAS39	<0.05
COL15A1	<0.05	KDM3B	<0.05	PTP4A3	<0.05	VIT	<0.01
COL17A1	<0.05	KDM4B	<0.05	PTPLAD2	<0.05	VOPP1	<0.005
COL18A1	<0.005	KDM4C	<0.005	PTPN11	<0.005	VPS13A	<0.01
COL19A1	<0.05	KDM4D	<0.01	PTPN12	<0.05	VPS13B	<0.001
COL21A1	<0.01	KDSR	<0.01	PTPN2	<0.01	VPS13C	<0.05
COL22A1	<0.005	KEL	<0.05	PTPN21	<0.005	VPS13D	<0.001
COL23A1	<0.05	KHDRBS2	<0.05	PTPN22	<0.05	VPS37A	<0.05
COL24A1	<0.05	KIAA0195	<0.05	PTPN23	<0.05	VPS41	<0.05
COL25A1	<0.001	KIAA0196	<0.01	PTPN3	<0.05	VPS52	<0.05
COL26A1	<0.05	KIAA0232	<0.05	PTPN5	<0.001	VPS53	<0.005

COL27A1	<0.05	KIAA0319	<0.05	PTPRA	<0.05	VSTM2A	<0.05
COL28A1	<0.01	KIAA0355	<0.001	PTPRB	<0.005	VSTM4	<0.005
COL4A1	<0.05	KIAA0368	<0.05	PTPRC	<0.05	VSX2	<0.005
COL4A2	<0.001	KIAA0513	<0.05	PTPRD	<0.001	VTI1A	<0.005
COL4A3	<0.05	KIAA0556	<0.005	PTPRE	<0.05	VWA3B	<0.001
COL4A4	<0.05	KIAA0753	<0.05	PTPRG	<0.001	VWA5B1	<0.005
COL5A1	<0.005	KIAA0895	<0.01	PTPRJ	<0.05	VWA7	<0.05
COL5A2	<0.05	KIAA0930	<0.005	PTPRK	<0.001	VWA8	<0.005
COL5A3	<0.05	KIAA0947	<0.05	PTPRM	<0.05	VWC2L	<0.05
COL6A1	<0.05	KIAA1109	<0.05	PTPRN2	<0.001	VWDE	<0.01
COL6A3	<0.005	KIAA1211	<0.05	PTPRO	<0.005	VWF	<0.005
COL7A1	<0.01	KIAA1217	<0.005	PTPRR	<0.005	WAPAL	<0.05
COL8A1	<0.05	KIAA1244	<0.05	PTPRT	<0.005	WBP1L	<0.05
COL9A1	<0.05	KIAA1257	<0.05	PTPRU	<0.05	WBSCR17	<0.001
COLEC12	<0.005	KIAA1324L	<0.001	PTPRZ1	<0.01	WDFY2	<0.05
COLGALT2	<0.05	KIAA1377	<0.05	PUM1	<0.05	WDFY3	<0.005
COLQ	<0.005	KIAA1432	<0.01	PURG	<0.05	WDFY4	<0.01
COMMD4	<0.05	KIAA1467	<0.01	PVALB	<0.01	WDPCP	<0.01
COPB1	<0.05	KIAA1468	<0.05	PWWP2A	<0.05	WDR1	<0.005
COPS7B	<0.05	KIAA1549	<0.05	PXDN	<0.05	WDR11	<0.05
COPZ1	<0.001	KIAA1551	<0.01	PXDNL	<0.005	WDR17	<0.05
COQ7	<0.01	KIAA1614	<0.05	PXK	<0.05	WDR18	<0.001
CORIN	<0.005	KIAA1683	<0.005	PXYLP1	<0.005	WDR27	<0.05
CORO2A	<0.01	KIAA1751	<0.05	PZP	<0.01	WDR33	<0.05
CORO2B	<0.05	KIAA1804	<0.05	QSOX1	<0.05	WDR36	<0.05
COX10	<0.001	KIAA1841	<0.05	R3HCC1L	<0.05	WDR49	<0.05
CP	<0.05	KIAA1958	<0.05	R3HDM1	<0.001	WDR60	<0.05
CPA2	<0.05	KIAA2026	<0.05	RAB11FIP1	<0.05	WDR61	<0.05
CPA6	<0.01	KIDINS220	<0.05	RAB11FIP4	<0.05	WDR62	<0.005
CPAMD8	<0.05	KIF11	<0.05	RAB27B	<0.005	WDR63	<0.05
CPB1	<0.05	KIF13A	<0.05	RAB3GAP1	<0.001	WDR64	<0.05
CPD	<0.001	KIF14	<0.01	RAB3IL1	<0.05	WDR65	<0.01
CPE	<0.01	KIF16B	<0.005	RAB40C	<0.05	WDR7	<0.05
CPEB2	<0.01	KIF17	<0.001	RABEP2	<0.05	WDR70	<0.05
CPED1	<0.005	KIF18A	<0.005	RAC2	<0.05	WDR72	<0.005
CPLX2	<0.05	KIF20B	<0.05	RAD21	<0.05	WDR74	<0.05
CPLX4	<0.05	KIF21A	<0.01	RAD21L1	<0.005	WFDC3	<0.05
CPN2	<0.05	KIF21B	<0.05	RAD50	<0.05	WFDC5	<0.05
CPNE3	<0.05	KIF26A	<0.05	RAD54B	<0.05	WFS1	<0.05
CPNE4	<0.001	KIF2A	<0.005	RADIL	<0.05	WHSC1	<0.05
CPPED1	<0.05	KIF3B	<0.05	RAF1	<0.005	WISP1	<0.001
CPS1	<0.01	KIF3C	<0.05	RAI1	<0.005	WNK1	<0.05
CPXM2	<0.05	KIF5C	<0.05	RAI14	<0.01	WNT10A	<0.05
CPZ	<0.05	KIF6	<0.001	RALGAPA2	<0.05	WNT2B	<0.05
CR1	<0.05	KIFAP3	<0.05	RALYL	<0.01	WNT5B	<0.05
CRB1	<0.005	KIN	<0.05	RAMP3	<0.05	WRN	<0.05
CREB3L3	<0.05	KIT	<0.005	RANBP17	<0.05	WSCD1	<0.01
CREB5	<0.01	KITLG	<0.001	RANBP2	<0.05	WSCD2	<0.001
CREBBP	<0.005	KL	<0.01	RANBP3L	<0.05	WWC1	<0.05
CREBL2	<0.05	KLB	<0.001	RANGAP1	<0.05	WWC2	<0.05

CRHR2	<0.05	KLC1	<0.05	RAP1A	<0.05	WWP1	<0.005
CRIM1	<0.05	KLF11	<0.005	RAPGEF2	<0.05	WWTR1	<0.005
CRISP1	<0.05	KLF17	<0.01	RAPGEF3	<0.05	XKR3	<0.05
CRISP3	<0.05	KLF7	<0.01	RAPGEF4	<0.005	XKR4	<0.005
CRISPLD2	<0.001	KLHDC4	<0.05	RAPGEF5	<0.05	XKR6	<0.05
CRMP1	<0.005	KLHDC8A	<0.05	RAPGEF6	<0.05	XPC	<0.05
CRP	<0.05	KLHL1	<0.01	RASA3	<0.05	XPNPEP1	<0.05
CRTAC1	<0.01	KLHL25	<0.05	RASAL2	<0.01	XPO1	<0.005
CRTC1	<0.05	KLHL32	<0.05	RASEF	<0.05	XPO4	<0.01
CRYBA4	<0.05	KLHL33	<0.05	RASGEF1C	<0.01	XPO5	<0.05
CRYBG3	<0.05	KLHL8	<0.05	RASGRF1	<0.005	XPO7	<0.05
CRYGN	<0.05	KLK2	<0.05	RASGRF2	<0.001	XPR1	<0.05
CSE1L	<0.01	KLK3	<0.05	RASGRP1	<0.05	XRCC1	<0.05
CSGALNACT1	<0.01	KLKB1	<0.05	RASL12	<0.05	XRCC3	<0.05
CSGALNACT2	<0.05	KMT2A	<0.005	RASSF1	<0.05	XRCC4	<0.05
CSMD1	<0.001	KNTC1	<0.05	RASSF5	<0.05	XRCC6BP1	<0.05
CSMD2	<0.005	KPTN	<0.05	RASSF9	<0.05	XRN1	<0.05
CSMD3	<0.01	KRAS	<0.005	RB1	<0.05	XYLB	<0.05
CSN1S1	<0.05	KRT23	<0.05	RB1CC1	<0.05	XYLT1	<0.05
CSNK1E	<0.05	KRT33A	<0.05	RBFOX1	<0.001	YEATS2	<0.005
CSPP1	<0.05	KRT34	<0.01	RBFOX2	<0.01	YES1	<0.05
CSRNP3	<0.05	KRT35	<0.005	RBKS	<0.05	YIPF5	<0.05
CSRP2BP	<0.05	KRT38	<0.05	RBL1	<0.01	YPEL5	<0.005
CSTA	<0.005	KRT5	<0.05	RBM19	<0.05	YTHDC2	<0.001
CTAGE5	<0.05	KRT6A	<0.05	RBM26	<0.05	ZAK	<0.01
CTBP2	<0.001	KRT6C	<0.05	RBM28	<0.01	ZBED4	<0.05
CTC1	<0.001	KRT71	<0.05	RBM33	<0.05	ZBTB16	<0.001
CTCFL	<0.05	KRT72	<0.05	RBM43	<0.01	ZBTB2	<0.05
CTIF	<0.005	KRT74	<0.005	RBM46	<0.05	ZBTB20	<0.005
CTNNA2	<0.005	KRT82	<0.005	RBM6	<0.05	ZBTB38	<0.005
CTNNA3	<0.001	KRT83	<0.05	RBMS3	<0.05	ZBTB4	<0.001
CTNND2	<0.01	KRT84	<0.005	RBP2	<0.05	ZBTB46	<0.01
CUBN	<0.001	KRT85	<0.001	RCAN1	<0.05	ZC2HC1B	<0.01
CUEDC1	<0.005	KRT86	<0.05	RCL1	<0.05	ZC3H13	<0.005
CUL1	<0.05	KRTAP4-2	<0.05	RCOR1	<0.01	ZC3H14	<0.05
CUL3	<0.01	KSR1	<0.01	RCSD1	<0.001	ZC3H15	<0.05
CUL5	<0.05	KSR2	<0.005	RDH13	<0.05	ZCCHC11	<0.05
CUL9	<0.05	KXD1	<0.05	RECK	<0.05	ZCCHC14	<0.005
CUX1	<0.01	KYNU	<0.01	REEP1	<0.05	ZCCHC6	<0.05
CUX2	<0.005	L1TD1	<0.05	REG1A	<0.01	ZCCHC7	<0.05
CXADR	<0.05	L3MBTL3	<0.001	REG1B	<0.005	ZDBF2	<0.05
CXCL16	<0.005	L3MBTL4	<0.05	REG4	<0.05	ZDHHC11	<0.05
CXCL3	<0.05	LAMA1	<0.05	RELN	<0.005	ZEB2	<0.01
CYB5R3	<0.05	LAMA2	<0.05	RERE	<0.005	ZFAND1	<0.005
CYB5R4	<0.05	LAMA3	<0.005	RERG	<0.05	ZFAND3	<0.01
CYFIP1	<0.005	LAMA4	<0.05	REST	<0.05	ZFAT	<0.05
CYFIP2	<0.005	LAMA5	<0.01	REV3L	<0.05	ZFHX3	<0.05
CYLC2	<0.005	LAMB1	<0.001	RFT1	<0.001	ZFHX4	<0.005
CYP17A1	<0.05	LAMB3	<0.05	RFWD2	<0.05	ZFP2	<0.05
CYP2C18	<0.05	LAMB4	<0.05	RFX8	<0.005	ZFP37	<0.001

CYP2C19	<0.01	LAMC3	<0.001	RGMA	<0.05	ZFP57	<0.05
CYP2C9	<0.005	LAMP3	<0.05	RGS12	<0.05	ZFP64	<0.005
CYP2R1	<0.05	LANCL2	<0.001	RGS22	<0.05	ZFPM2	<0.005
CYP46A1	<0.05	LARGE	<0.005	RGS6	<0.005	ZFR	<0.05
CYP4A11	<0.05	LARP1	<0.01	RGS7	<0.01	ZFYVE1	<0.005
CYP4B1	<0.05	LARS2	<0.05	RGS7BP	<0.01	ZFYVE28	<0.05
CYP4F11	<0.05	LATS2	<0.05	RGS9	<0.05	ZFYVE9	<0.01
CYP4F12	<0.05	LBR	<0.05	RGSL1	<0.05	ZGPAT	<0.05
CYP4F22	<0.005	LCAT	<0.05	RHAG	<0.05	ZGRF1	<0.05
CYP4F3	<0.05	LCLAT1	<0.05	RHBDD2	<0.005	ZHX2	<0.05
CYP4X1	<0.05	LCN15	<0.05	RHBDF1	<0.05	ZIM3	<0.05
CYP7B1	<0.01	LCP1	<0.05	RHOJ	<0.005	ZMAT4	<0.01
CYTIP	<0.05	LCT	<0.001	RHPN2	<0.05	ZMAT5	<0.005
DAAM2	<0.05	LDB2	<0.005	RIC3	<0.001	ZMIZ1	<0.01
DAB1	<0.005	LDLR	<0.01	RICTOR	<0.05	ZMYM6	<0.05
DAB2IP	<0.05	LECT1	<0.05	RIF1	<0.05	ZMYND15	<0.05
DACH1	<0.05	LEPR	<0.05	RIMBP2	<0.01	ZNF10	<0.05
DAPK1	<0.05	LEPREL1	<0.001	RIMS1	<0.05	ZNF101	<0.05
DAPK2	<0.05	LEPREL2	<0.05	RIMS2	<0.01	ZNF106	<0.01
DCAF11	<0.05	LGI1	<0.05	RIT2	<0.05	ZNF112	<0.05
DCAF17	<0.05	LGI2	<0.05	RMND5A	<0.05	ZNF114	<0.05
DCAF4	<0.001	LGR5	<0.05	RNF123	<0.05	ZNF132	<0.05
DCAF4L1	<0.01	LGR6	<0.01	RNF145	<0.05	ZNF133	<0.05
DCAF6	<0.05	LHFPL4	<0.01	RNF150	<0.05	ZNF148	<0.05
DCBLD1	<0.05	LHFPL5	<0.005	RNF169	<0.05	ZNF160	<0.01
DCBLD2	<0.05	LIFR	<0.05	RNF17	<0.05	ZNF169	<0.05
DCC	<0.005	LIG3	<0.05	RNF19A	<0.05	ZNF180	<0.01
DCDC1	<0.01	LILRA1	<0.005	RNF212	<0.005	ZNF181	<0.05
DCDC5	<0.05	LILRA2	<0.01	RNF217	<0.05	ZNF208	<0.001
DCHS1	<0.05	LIMCH1	<0.005	RNF220	<0.05	ZNF215	<0.05
DCHS2	<0.05	LIN7A	<0.005	RNF26	<0.05	ZNF217	<0.01
DCLK1	<0.005	LINGO2	<0.005	RNF43	<0.005	ZNF226	<0.05
DCLK2	<0.001	LIPC	<0.005	RNF8	<0.05	ZNF227	<0.01
DCP1A	<0.05	LIPG	<0.05	RNPEP	<0.05	ZNF233	<0.001
DDAH1	<0.05	LIPK	<0.05	ROBO1	<0.001	ZNF250	<0.05
DDC	<0.05	LIX1	<0.05	ROBO2	<0.001	ZNF256	<0.05
DDHD1	<0.05	LMLN	<0.05	ROBO4	<0.05	ZNF257	<0.001
DDR2	<0.005	LMO7	<0.05	ROCK1	<0.05	ZNF267	<0.05
DDX21	<0.05	LMOD1	<0.05	ROPN1	<0.01	ZNF280D	<0.05
DDX27	<0.05	LMTK2	<0.01	ROR1	<0.01	ZNF285	<0.01
DDX42	<0.05	LMX1A	<0.05	ROR2	<0.05	ZNF292	<0.05
DDX46	<0.001	LOX	<0.05	RORB	<0.005	ZNF300	<0.05
DDX58	<0.05	LOXHD1	<0.005	RORC	<0.01	ZNF331	<0.05
DDX60	<0.01	LOXL2	<0.05	ROS1	<0.05	ZNF335	<0.05
DDX60L	<0.005	LPA	<0.01	RPE65	<0.05	ZNF33A	<0.005
DECR1	<0.01	LPAL2	<0.05	RPGRIP1L	<0.05	ZNF34	<0.05
DEF8	<0.05	LPAR1	<0.005	RPH3A	<0.005	ZNF362	<0.05
DEGS2	<0.05	LPAR3	<0.01	RPH3AL	<0.005	ZNF365	<0.05
DENND1B	<0.001	LPCAT1	<0.05	RPN2	<0.001	ZNF366	<0.005
DENND2D	<0.05	LPGAT1	<0.001	RPRD2	<0.005	ZNF383	<0.05

DENND3	<0.05	LPHN2	<0.05	RPS6KA2	<0.005	ZNF385D	<0.005
DENND6A	<0.005	LPHN3	<0.005	RPTOR	<0.005	ZNF407	<0.001
DEPDC1	<0.01	LPIN1	<0.05	RREB1	<0.005	ZNF410	<0.05
DEPDC5	<0.05	LPP	<0.001	RRM1	<0.01	ZNF423	<0.01
DFNB31	<0.05	LPPR1	<0.005	RRP12	<0.01	ZNF429	<0.05
DGCR2	<0.05	LPPR4	<0.05	RSF1	<0.05	ZNF43	<0.05
DGKB	<0.001	LRBA	<0.001	RSPH3	<0.05	ZNF438	<0.01
DGKI	<0.01	LRCH1	<0.05	RSPH9	<0.01	ZNF454	<0.05
DHCR7	<0.05	LRCH3	<0.05	RSPO2	<0.01	ZNF462	<0.05
DHDH	<0.05	LRFN2	<0.05	RSPO4	<0.01	ZNF471	<0.05
DHX29	<0.05	LRFN5	<0.001	RSRC1	<0.05	ZNF485	<0.005
DHX57	<0.005	LRGUK	<0.005	RTCB	<0.05	ZNF507	<0.05
DHX8	<0.05	LRIG1	<0.005	RTN4	<0.05	ZNF516	<0.05
DHX9	<0.05	LRIT1	<0.01	RUNX1T1	<0.05	ZNF519	<0.01
DIAPH1	<0.005	LRMP	<0.01	RUNX2	<0.05	ZNF532	<0.05
DIAPH3	<0.01	LRP1	<0.05	RUSC2	<0.01	ZNF536	<0.005
DIDO1	<0.05	LRP12	<0.005	RXFP1	<0.05	ZNF544	<0.05
DIEXF	<0.05	LRP1B	<0.001	RXFP2	<0.01	ZNF568	<0.05
DIMT1	<0.05	LRP2	<0.01	RYR1	<0.01	ZNF582	<0.05
DIO3	<0.05	LRP4	<0.05	RYR2	<0.01	ZNF585A	<0.05
DIP2C	<0.001	LRP5	<0.05	RYR3	<0.01	ZNF585B	<0.05
DIRC2	<0.005	LRP8	<0.05	SACS	<0.005	ZNF608	<0.005
DKK2	<0.005	LRPAP1	<0.01	SALL1	<0.05	ZNF609	<0.05
DKKL1	<0.05	LRPPRC	<0.005	SALL2	<0.05	ZNF610	<0.05
DLC1	<0.01	LRRC14B	<0.05	SALL4	<0.001	ZNF623	<0.05
DLG2	<0.001	LRRC20	<0.05	SAMD12	<0.01	ZNF626	<0.01
DLGAP1	<0.001	LRRC31	<0.001	SAMD14	<0.05	ZNF627	<0.05
DLGAP2	<0.001	LRRC36	<0.05	SAMD15	<0.05	ZNF638	<0.01
DLGAP3	<0.05	LRRC47	<0.05	SAMD3	<0.005	ZNF665	<0.05
DLGAP4	<0.05	LRRC49	<0.005	SAMD4B	<0.05	ZNF676	<0.001
DLK1	<0.01	LRRC4C	<0.005	SAMD7	<0.01	ZNF677	<0.05
DLL3	<0.05	LRRC7	<0.01	SAMD9	<0.05	ZNF678	<0.01
DMGDH	<0.05	LRRC71	<0.001	SAMHD1	<0.001	ZNF679	<0.005
DMKN	<0.05	LRRC8C	<0.05	SAMSN1	<0.05	ZNF681	<0.05
DMXL2	<0.05	LRRFIP1	<0.01	SAP130	<0.05	ZNF708	<0.05
DNAH10	<0.005	LRRIQ1	<0.05	SARS2	<0.05	ZNF71	<0.05
DNAH11	<0.005	LRRIQ3	<0.005	SASH1	<0.005	ZNF720	<0.05
DNAH12	<0.005	LRRIQ4	<0.001	SASS6	<0.05	ZNF721	<0.01
DNAH17	<0.05	LRRK1	<0.05	SATB1	<0.05	ZNF727P	<0.05
DNAH3	<0.005	LRRK2	<0.01	SATB2	<0.001	ZNF766	<0.05
DNAH5	<0.005	LRRN1	<0.05	SBF2	<0.01	ZNF777	<0.01
DNAH6	<0.005	LRRN4	<0.05	SBNO1	<0.001	ZNF786	<0.05
DNAH8	<0.01	LSG1	<0.005	SBSN	<0.05	ZNF8	<0.05
DNAH9	<0.05	LTBP1	<0.01	SCARA5	<0.005	ZNF804A	<0.01
DNAJB1	<0.05	LTBP2	<0.005	SCFD2	<0.005	ZNF804B	<0.001
DNAJC1	<0.05	LUC7L2	<0.05	SCGN	<0.05	ZNF805	<0.05
DNAJC13	<0.01	LUZP2	<0.01	SCMH1	<0.05	ZNF812	<0.05
DNAJC5B	<0.05	LYST	<0.005	SCN10A	<0.05	ZNF821	<0.05
DNAJC7	<0.005	MACF1	<0.05	SCN1A	<0.05	ZNF827	<0.005
DNER	<0.005	MACROD2	<0.001	SCN2A	<0.005	ZNF831	<0.01

DNHD1	<0.005	MAEA	<0.05	SCN2B	<0.05	ZNF836	<0.05
DNM2	<0.05	MAEL	<0.05	SCN7A	<0.05	ZNF880	<0.05
DNM3	<0.01	MAGI1	<0.001	SCN8A	<0.05	ZNF92	<0.05
DNMT1	<0.05	MAGI2	<0.001	SCN9A	<0.001	ZNRF3	<0.001
DNMT3B	<0.05	MAGI3	<0.05	SCNN1B	<0.005	ZNRF4	<0.05
DNTT	<0.001	MAML2	<0.001	SCNN1G	<0.01	ZPBP	<0.01
DOCK1	<0.005	MAML3	<0.05	SCRN1	<0.001	ZPR1	<0.05
DOCK10	<0.01	MAN1A1	<0.05	SCRN3	<0.05	ZRANB3	<0.05
DOCK2	<0.005	MAN1C1	<0.05	SCTR	<0.05	ZSCAN1	<0.05
DOCK3	<0.05	MAN2A1	<0.05	SCUBE1	<0.005	ZSCAN2	<0.05
DOCK4	<0.005	MANEAL	<0.05	SCUBE2	<0.005	ZSCAN29	<0.05
DOCK5	<0.05	MAP10	<0.01	SCUBE3	<0.01	ZSCAN4	<0.001
DOCK6	<0.05	MAP1A	<0.05	SDHA	<0.01	ZSWIM5	<0.05
DOCK8	<0.001	MAP1B	<0.05	SDK1	<0.005	ZW10	<0.001
DOCK9	<0.05	MAP2	<0.01	SDK2	<0.005	ZYG11A	<0.05
DOK6	<0.005	MAP2K1	<0.05	SDPR	<0.05	ZZEF1	<0.05
DOK7	<0.005	MAP2K2	<0.005	SDR16C5	<0.05		
DOPEY2	<0.05	MAP2K6	<0.05	SEC16A	<0.05		
